# Nanozymes Empower Periodontitis Treatment: New Strategies and Clinical Application Prospects

**DOI:** 10.34133/bmr.0210

**Published:** 2025-05-20

**Authors:** Yurong Xu, Jingyu Yan, Chenying Cui, Lihong Zhou, Kaifang Zhang, Meijun Du, Yajuan Gong, Zhuowei Zhang, Xiuping Wu, Bing Li

**Affiliations:** ^1^ Shanxi Medical University School and Hospital of Stomatology, Taiyuan 030001, Shanxi, China.; ^2^ Shanxi Province Key Laboratory of Oral Diseases Prevention and New Materials, Taiyuan 030001, Shanxi, China.; ^3^ Academy of Medical Sciences, Shanxi Medical University, Taiyuan 030001, Shanxi, China.

## Abstract

Periodontitis is a chronic inflammatory disease mediated by the immune system. Its pathogenesis involves the interaction of multiple factors, among which the accumulation of dental plaque is considered the initial key factor in the onset of the disease. As the pathogenic bacteria in plaque proliferate and metabolites are released, the host’s immune system produces a strong response, leading to an inflammatory response and structural destruction of local tissues. Traditional treatment relies on mechanical scraping and antibiotics but suffers from tissue damage, difficulty in removing deep-seated bacteria, development of drug resistance, and insufficient modulation of complex pathomechanisms. Nanozymes, as a novel therapeutic tool with high efficiency, stability, and multifunctionality, can remove pathogenic bacteria, modulate inflammation, and promote tissue repair, as well as have better environmental stability and biocompatibility, which provides a new way for precise treatment of periodontal disease and tissue regeneration. In this paper, the pathophysiology of periodontitis was first elucidated, and the design strategy of nanozymes and their application classification for the treatment of periodontitis were also discussed. Then, the recent advances in treating periodontitis with nanozymes are summarized in terms of antibacterial, anti-inflammatory, and tissue regeneration. Finally, the problems and prospects for the development of nanozymes for the treatment of periodontitis are discussed in terms of current challenges in the treatment of periodontitis and the stimulation of innovative research on nanozymes drugs, with a view to the clinical translation of novel enzyme mimicry strategies and efficient nanozymes for periodontitis drugs.

## Introduction

Periodontitis is a prevalent chronic inflammatory disease of the oral cavity. Its clinical manifestations include bleeding and receding gums, tooth displacement, and alveolar bone resorption [1]. If treatment is not timely or appropriate, it may cause loosening and loss of teeth, affecting aesthetic appearance and, in severe cases, leading to impaired chewing function, and may even lead to mental health problems [2]. In China, periodontal disease is more prevalent than dental caries across all age groups, with severe cases posing a substantial public health challenge. In addition, the close and complex association between periodontitis and other diseases such as cardiovascular disease, diabetes mellitus, autoimmune disorders, rheumatoid arthritis, and poor pregnancy outcomes not only places a burden on patients but also puts a marked economic strain on the healthcare system [3–5]. Therefore, early diagnosis and intervention of periodontitis is crucial for maintaining oral health and improving quality of life. With the increasing research on periodontitis, it is widely recognized that the destruction of periodontal tissues is due to a combination of plaque biofilm and an imbalance of host immune response. Under the continuous stimulation of pathogenic bacteria, periodontal tissues will release many inflammatory mediators and reactive oxygen species (ROS), destroying the balance of the periodontal microenvironment and leading to the damage of periodontal soft and hard tissue [6]. Therefore, most patients with periodontitis need to undergo basic treatments such as scaling and root planing to remove dental plaque [7]. However, existing studies have revealed that simple scaling and root planing cannot efficiently remove pathogenic microorganisms in periodontal tissues, especially those in areas that are difficult for treatment instruments to reach, as well as those that have already invaded the periodontal tissue [8]. To enhance the therapeutic outcomes, pharmacological treatments have become routine approaches, including local drug delivery, systemic antibiotic therapy, and the use of systemic host modulators [9]. Local drug delivery may face the issue of rapid drug release, resulting in a low local absorption rat [10]. Prolonged use of antibiotics can lead to bacterial resistance, reducing or even nullifying the effectiveness of antibiotics in future treatments [11]. The use of systemic host modulators, on the other hand, increases the risk of adverse reactions and drug interactions in patients [12]. Given these challenges, there is an urgent need to explore innovative therapeutic strategies to enhance the management of periodontitis. The latest advancements in nanotechnology, particularly the innovation of nanozymes, offer new solutions to address the challenges posed by periodontitis. Compared to traditional materials, nanozymes have demonstrated extensive promise in the therapy and management of periodontitis, mainly in the following 4 aspects: (a) Enzyme activity simulation: Nanozymes can simulate the peroxidase (POD) or oxidase (OXD) activity of natural enzymes. By catalyzing the production or scavenging of ROS, they can disrupt the cell walls or membranes of pathogens, or interfere with their metabolic pathways, thereby inhibiting the growth of bacteria on the surface and deep within the periodontal tissues. This effectively avoids the tissue damage that may be caused by mechanical debridement [13,14]. (b) Inflammation regulation: Nanozymes can modulate the transition of macrophages from the M1 to the M2 phenotype, reduce the expression of pro-inflammatory cytokines, and simultaneously increase the production of anti-inflammatory cytokines, thereby effectively alleviating the inflammatory response in periodontitis [15–17]. (c) Targeted drug delivery: Nanozymes can be engineered with surface modifications to create targeted drug delivery systems. These systems can specifically recognize and bind to biomarkers associated with periodontitis, delivering therapeutic agents directly to the affected sites. This targeted delivery approach enhances therapeutic efficiency, reduces damage to normal cells, and minimizes drug side effects [18]. (d) Cost-effectiveness and scalability: The synthesis of nanozymes can utilize cost-effective approaches and materials to enhance their feasibility for extensive manufacturing and clinical application. The cost-effectiveness of nanozymes, combined with their high efficiency and targeting ability, makes them a strong candidate for the treatment of periodontitis and holds promise for important roles in future clinical applications [19]. These characteristics indicate that nanozymes have broad prospects in the handling and therapy of periodontitis, offering the potential to provide patients with safer and more effective therapeutic options.

**Fig. 1.**
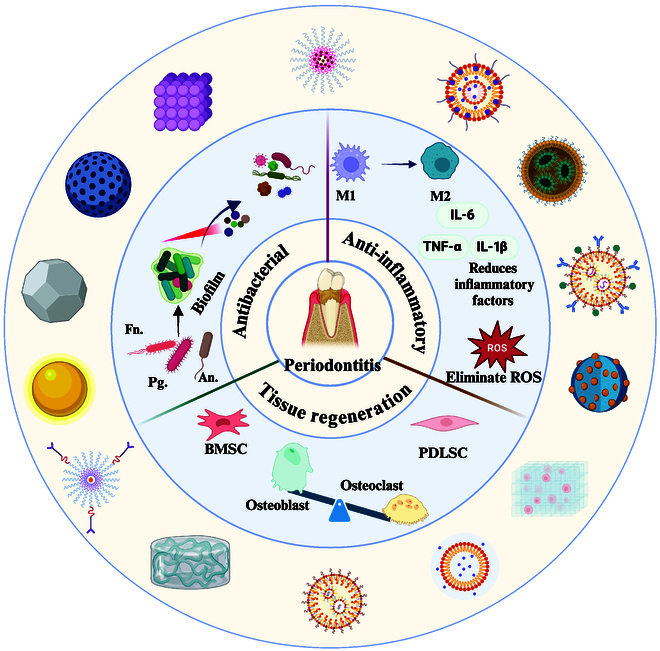
Schematic overview of nanozyme-based therapeutic agents via triple concerto of antimicrobial activity, anti-inflammatory effects, and periodontium regeneration.

Although numerous studies have demonstrated the potential of nanozymes in the control of periodontitis, there is a lack of comprehensive classification and review on the physicochemical properties of nanozymes, their therapeutic mechanisms in treating periodontitis, and the progress of their applications. Recent investigations have predominantly concentrated on the synthesis and progression of nanozymes, emphasizing their drug-carrying capacity and catalytic activity, while relatively few reviews on the physicochemical properties of nanozymes and their therapeutic mechanisms for the treatment of periodontitis have been conducted, lacking a systematic evaluation and a comprehensive summary. With the continuous increase in the literature related to the treatment of periodontitis by nanozymes in recent years, we believe that it is necessary to conduct a comprehensive review of the current research progress, to offer a theoretical foundation and practical insights for the broad future application of nanozymes in periodontitis treatment (Fig. 1). Therefore, this article provides a systematic review that comprehensively examines the mechanisms of nanozymes in the treatment of periodontitis and their latest advances in biomedical applications.

First, we introduced the pathogenesis of periodontitis. Subsequently, the design ideas of nanozymes are summarized, which are mainly introduced in 3 aspects: enzyme mimetic activity, size and shape regulation, and surface engineering. We also summarized the research progress of different kinds of nanozymes in periodontitis in recent years based on the latest research results and discussed the mechanism of nanozymes for periodontitis in depth. On this basis, this paper further looks into the current challenges and prospects, aiming to stimulate the development of new strategies and facilitate the clinical translation of more effective nanozymatic drugs.

## The Pathogenesis of Periodontitis

**Fig. 2.**
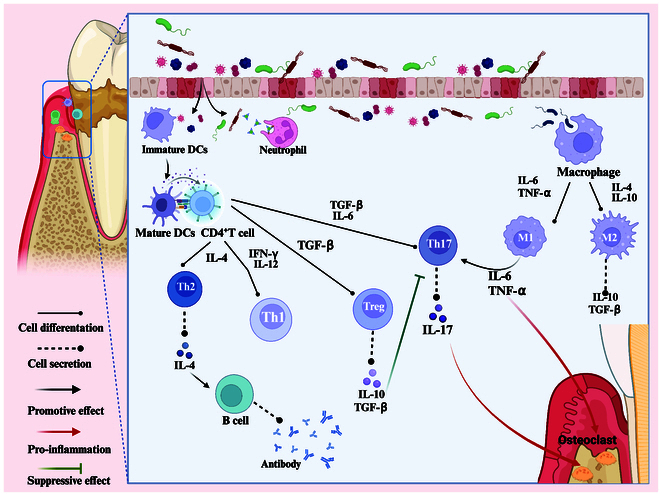
The pathogenesis of periodontitis.

As research on periodontitis deepens, scientists are gradually revealing its complex pathogenesis, which involves genetic factors, plaque biofilm formation, and aberrant activation of the host’s immune response. In order to treat periodontitis more effectively, it is crucial to understand its pathogenesis (Fig. 2).

Under normal physiological conditions, a bacterial community exists around the periodontal tissues, and these microorganisms maintain a symbiotic equilibrium with the host’s immune system, thus avoiding disruptions in the periodontal ecosystem [20]. However, in the pathological state of periodontitis, pathogenic microorganisms upset this balance, triggering an excessive immune response, which subsequently results in damage to the soft and hard structures of the periodontal tissue [21]. Metabolites from tissue destruction provide nutrients for pathogenic bacteria, further exacerbating the microecological imbalance and creating a vicious cycle that continues to promote the progression of periodontal disease. Although the exact pathogenesis of periodontitis is not yet fully understood, available studies have revealed the key roles of genetic factors and plaque biofilms in the pathogenesis. The major periodontal pathogens include *Porphyromonas gingivalis*, *Fusobacterium nucleatum*, *Actinobacillus actinomycetemcomitans*, *Prevotella intermedia*, and *Treponema denticol* [22]. Initially, proteins or glycoproteins from saliva are adsorbed to the tooth surface, forming a film called the acquired film. Subsequently, bacteria in the oral cavity begin to colonize the film one after another, with different species recognizing and adhering to each other through intermolecular specificity on the surface. Bacteria connect with each other through adhesion and copolymerization and rapidly divide, multiply, and grow to form a complex ecological community. In addition, pathogenic bacteria synthesize extracellular polysaccharides from sucrose by secreting extracellular enzymes, a process that facilitates their adhesion to the tooth surface, which in turn accelerates localized aggregation of microorganisms and builds the complex network structure of the biofilm matrix [23]. A deeper understanding of these complex pathogenic mechanisms may pave the way for novel therapeutic strategies against periodontitis, particularly by controlling biofilms on teeth. It is important to recognize that plaque biofilms are not the sole determinant of periodontal pathology. Although bacterial infection serves as a critical precondition for the progression of periodontitis, the immune response of the host plays a crucial role in the progression of periodontitis and tissue destruction [24]. As the front line of the nonspecific immune response, neutrophils are responsible for phagocytosis and elimination of pathogens, but in periodontitis, they are often overwhelmed by the presence of pathogens in large numbers [25]. Dendritic cells (DCs) play the role of a bridge between innate and adaptive immune responses in periodontitis. Immature DCs exhibit potent phagocytic activity and can rapidly engulf invading pathogens [26]. When naive CD4^+^ helper T cells (TH0) interact with mature DCs, they differentiate into various T cell subsets, including TH1, TH2, TH17, and regulatory T cells (Treg), based on the cytokine signals they receive [27]. The differentiation process of these T cell subsets is determined by the specific cytokine environment. In the inflammatory environment, interferon-γ (IFN-γ) and interleukin-12 (IL-12) secreted by DCs promote the differentiation and formation of TH1 cells [28]. Studies have shown that TH1 cells play a role in the formation of osteoclasts and the loss of alveolar bone [29]. On the other hand, TH2 cells are induced to differentiate by IL-4, and the IL-4 secreted by these TH2 cells can further activate B cells and promote the production of antibodies [30]. The other 2 CD4^+^ T cell subsets, TH17 and Treg, play crucial roles in the development of autoimmune diseases and in maintaining immune system homeostasis [31]. Under the combined influence of transforming growth factor-β (TGF-β), IL-1β, and IL-6, the TH17 cell subset is capable of secreting IL-17, IL-23, IL-22, IL-6, and tumor necrosis factor-α (TNF-α) [32]. IL-17 can stimulate the production of various inflammatory mediators, including TNF-α, prostaglandin E_2_ (PGE_2_), IL-6, and IL-1β [33]. These mediators promote bone resorption by activating osteoclasts. When TGF-β is present alone, Treg cells are induced to form and release the immunosuppressive cytokines IL-10 and TGF-β. This mechanism can inhibit the immune response of TH17 cells and is crucial for sustaining immune homeostasis [31,34]. At the same time, in the case of immune disorder, the aggregation of macrophages and their polarization toward the M1 phenotype will also be promoted [35]. M1 macrophages exacerbate inflammation by secreting monocyte chemoattractant protein, IL-6*,* TNF-α, and ROS [36]. In addition, M1 macrophages may also indirectly lead to bone tissue damage by influencing TH17 cells [37]. Similarly, the progression of periodontitis also involves the destruction of periodontal tissues such as the gingiva, cementum, and periodontal ligament (PDL). The cumulative effect of these destructive actions collectively promotes the deterioration of periodontitis.

Meanwhile, there exists a bidirectional and intricate pathological interaction between aging and periodontitis. With advancing age, the host’s immune response to periodontal pathogens becomes compromised, disturbing oral microbial homeostasis and facilitating persistent inflammation [38]. The phenomenon of “inflammaging”, which is a state of chronic, low-grade systemic inflammation marked by elevated cytokines such as IL-6 and TNF-α, intensifies periodontal tissue sensitivity to microbial challenges. Cellular senescence contributes to the progression of periodontitis, primarily through the release of senescence-associated secretory phenotype (SASP) factors [39]. These secretions, which include pro-inflammatory mediators, chemokines, and matrix-degrading enzymes, amplify local inflammatory responses, impair tissue integrity, and hinder effective regeneration. Additionally, the paracrine actions of SASP promote senescence in surrounding healthy cells, thereby reinforcing a self-perpetuating loop of inflammation and cellular aging within periodontal tissues. On the other hand, periodontitis itself, being a chronic and sustained inflammatory disorder, can in turn accelerate both local and systemic aging processes [40]. Oxidative stress, inflammatory cytokines, and microbial toxins prevalent in periodontitis can lead to genomic instability, including DNA damage, telomere attrition, and epigenetic alterations, thereby driving premature cellular senescence. Moreover, the spread of inflammatory mediators beyond the periodontal environment may exacerbate systemic aging and elevate susceptibility to age-associated diseases.

In conclusion, the pathogenesis of periodontitis involves a dynamic interplay between dysbiotic microbial biofilms and the host immune system, where an excessive immune response leads to progressive destruction of periodontal tissues. The disease process is further complicated by aging, which weakens immune defenses and promotes chronic inflammation through mechanisms such as inflammaging and SASP. Moreover, periodontitis not only accelerates local tissue degeneration but also contributes to systemic aging via oxidative stress and pro-inflammatory signaling. Understanding these complex mechanisms is essential for developing more effective therapeutic strategies that target both microbial control and immune modulation in the management of periodontitis.

## Advances in Periodontal Treatment Strategies

Periodontal treatment strategies have been continuously refined over time, evolving into a comprehensive framework that integrates basic therapies with advanced interventions.

Basic periodontal treatments, such as mechanical scaling and root planning (SRP), are essential for controlling infections and slowing the progression of disease [9]. Mechanical scaling utilizes ultrasonic or manual instruments to remove plaque and calculus from teeth, while root planing smoothes the root surface to eliminate toxins and necrotic cementum, thereby facilitating reattachment of periodontal tissue. These procedures are cost-effective and provide immediate improvements in periodontal health. However, they have limitations in complex anatomical areas like furcation regions and deep pockets, where complete biofilm removal is challenging [8]. Moreover, without maintenance therapy, long-term outcomes may be compromised. Nonetheless, basic therapy remains essential for all subsequent advanced treatments. Nonsurgical methods, such as laser therapy and photodynamic therapy (PDT), have gained importance for mild to moderate periodontitis. Laser therapy reduces pocket depths and promotes healing through its thermal and biostimulatory effects, although its efficacy depends on the operator’s skill and the laser’s penetration [41]. PDT utilizes photosensitizers activated by specific wavelengths to produce ROS that target pathogens, offering targeted treatment with a low risk of drug resistance [42]. However, PDT’s effectiveness is limited by light penetration and photosensitizer distribution, and the procedure is relatively complex. For moderate to severe periodontal lesions, surgical interventions like flap surgery and bone grafting are crucial [43]. Flap surgery allows direct debridement of deep lesions, improving the local tissue environment. Bone grafting, using various bone materials, promotes alveolar bone regeneration and enhances tooth stability. However, these surgical treatments are invasive, have long recovery periods, and demand high technical skills [44].

The advent of biomaterials and nanotechnology has revolutionized periodontal therapy [45]. Nanozymes, nanomaterials with enzyme-like activity, are emerging as a promising new approach [13,14]. They mimic natural antioxidant enzymes to neutralize excess ROS, reducing inflammation and oxidative stress. Some nanozymes also have antibacterial properties, targeting periodontal pathogens. Their biocompatibility and ability to achieve targeted delivery and sustained release make them highly effective. Unlike traditional drug-delivering nanoparticles, nanozymes actively participate in regulating pathological processes, reducing drug degradation and side effects [46]. Compared to anti-inflammatory biomaterials, they offer greater reactivity in complex inflammatory environments [47]. Unlike laser or PDT, nanozymes do not require external activation and can function continuously in deep tissues [48]. Compared to surgery, nanozyme-based treatments are less invasive and simpler, making them suitable for various disease stages [49].

In summary, periodontal treatment now encompasses a multi-level system that includes basic therapies, minimally invasive interventions, surgical reconstruction, and biological targeting. Basic therapy remains the cornerstone for infection control, while nonsurgical methods provide supplementary support. Surgical treatments address complex lesions, and nanozymes offer new possibilities for precise inflammation control and tissue regeneration. Looking ahead, as our understanding of periodontal pathology deepens and materials science advances, nanozymes are poised to enhance personalized and precision periodontal therapy.

## Strategies for the Design of Nanozymes

Nanozymes are often regarded as an enchanting yet mysterious “black box”, with many of their catalytic mechanisms still shrouded in mystery. However, recent studies have gradually unveiled the secrets of nanozymes. It has been found that the catalytic efficiency of nanozymes is highly associated with their size, shape, surface modification, and other factors [50]. Although current research and applications on modulating the catalytic properties of nanozymes are primarily focused on the treatment of other disease, their application in the treatment of periodontitis also holds important research value. Through rational design, nanozymes can achieve precise and efficient therapeutic effects targeting the pathological features of periodontitis, such as pathogen infection, chronic inflammation, and oxidative stress.

### Enzyme-mimicking activity

**Fig. 3.**
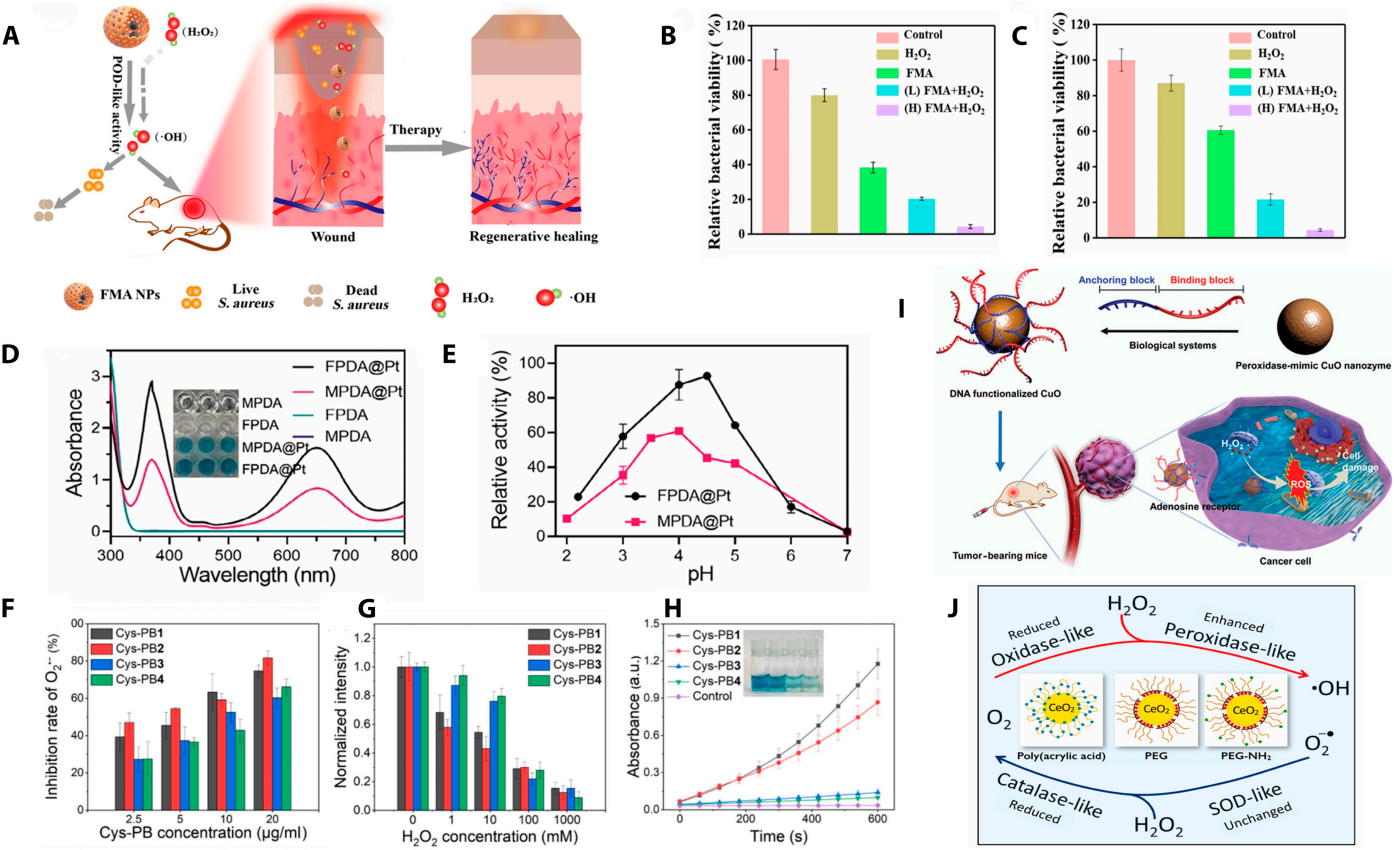
(A) Antibacterial wound-healing mechanism of FMA nanozymes via high in vivo peroxidase-like activity. (B) Extracellular antibacterial activity of FMA nanozymes against *E. coli*. (C) Antibacterial activity of FMA nanozymes against *S. aureus* in vitro. (A to C) Reproduced with permission [53]. Copyright 2023, Springer Nature. (D) Investigating the POD activity of different catalysts, such as MPDA, FPDA, MPDA@Pt, and FPDA@Pt. (E) Investigate the POD activity of MPDA@Pt and FPDA@Pt under various pH conditions. (D and E) Reproduced with permission [55]. Copyright 2024, John Wiley and Sons. (F) The inhibition rate of O_2_^−^ evaluated the SOD-like activity of Cys-PB. (G) The CAT-like activity of Cys-PB was determined by monitoring the fluorescence emission of [Ru(dpp)_3_] Cl_2_. (H) The POD-like activity of Cys-PB was assessed by monitoring the oxidation of TMB using ultraviolet–visible spectroscopy. (F to H) Reproduced with permission [59]. Copyright 2025, Elsevier. (I) Schematic of di-DNA-functionalized CuO nanozyme for in vivo targeted antitumor therapy. Reproduced with permission [62]. Copyright 2021, The Royal Society of Chemistry. (J) Modification of ceria with polymer coatings containing poly (ethylene glycol) and phosphonic acid groups can reduce, maintain, or enhance the enzyme-like catalytic activity. Reproduced with permission [63]. Copyright 2020, ACS Publications.

Nanozymes can replicate the activities of a wide range of natural enzymes, such as superoxide dismutase (SOD), catalase (CAT), OXD, and POD [51]. Among them, nanozymes with POD-like activity can catalyze the generation of highly toxic hydroxyl radicals (·OH) from hydrogen peroxide (H_2_O_2_). These radicals are capable of directly disrupting dental plaque biofilms, demonstrating potential in the antimicrobial treatment of periodontitis [52]. Liu et al. [53] developed a novel hybrid nanozyme, which involves the in situ growth of ultrasmall gold nanoparticles (Au NPs) on the surface of Fe_3_O_4_ nanoparticles stabilized by metal-organic frameworks (MOFs), forming Fe_3_O_4_@MOF@Au nanoparticles (FMA NPs). These nanozymes exhibit synergistic POD-like activity (Fig. 3A), enabling the efficient generation of a large number of ·OH in the presence of trace amounts of H_2_O_2_. Moreover, the FMA nanozymes show strong antibacterial effects against a variety of bacteria, including both Gram-negative bacteria (such as *Escherichia coli*) and Gram-positive bacteria (such as *Staphylococcus aureus*) (Fig. 3B and C).

In addition, patients with periodontitis often experience a notable increase in oxidative stress levels, which can damage periodontal tissues and exacerbate inflammatory responses. By designing nanozymes with SOD or CAT activity, excess ROS can be efficiently scavenged, thereby effectively alleviating the damage to periodontal tissues caused by oxidative stress. To overcome this obstacle, Gao et al. [54] created an innovative copper nanodot-based ionic gel (Cu-NDs/IL gel), which not only effectively penetrates tissues but also possesses antibacterial and anti-inflammatory properties. The gel was produced by integrating copper nanodots (Cu-NDs) with triple enzymatic activity into a multifunctional gel. The excellent antimicrobial effect of the Cu-NDs/IL gel is mainly due to the POD-like activity of the Cu-NDs, which also mimics the activities of SOD and CAT. This enables the gel to effectively scavenge free radicals in neutral environments, thereby reducing periodontal inflammation. Importantly, the ionic liquid (IL) within the gel facilitates the profound infiltration of Cu-NDs into gingival tissues, thereby enabling the full exploitation of Cu-NDs’ triple enzymatic activities. Overall, the Cu-NDs/IL gel demonstrates extensive promise as a topical therapeutic for periodontitis, enhancing drug delivery, eliminating bacteria, and neutralizing ROS.

### Design of size and shape

Nanozymes, as an emerging class of biocatalysts, show promising applications in the field of periodontitis treatment by their size- and shape-dependent unique catalytic properties. The size reduction substantially increases not only the specific surface area of nanozymes but also the number of active sites, thus improving the catalytic efficiency. This property is particularly important for periodontitis treatment, as nanozymes are required to efficiently scavenge excess ROS at the lesion site to alleviate oxidative stress and inflammatory responses. In addition, nanozymes with different morphologies (e.g., flower-like, spherical, or sheet-like structures) exhibit notable performance differences in catalytic reactions, which are mainly concentrated on the surface of the nanomaterials, due to the differences in their surface properties and the distribution of active sites. In periodontitis treatment, by optimizing the morphology characteristics of nanozymes, not only their antioxidant and anti-inflammatory properties can be improved, but also their tissue permeability and targeting at the lesion site can be enhanced. Xu et al. [55] meticulously engineered and systematically evaluated 2 distinct polydopamine/platinum (PDA@Pt) nanozymes—namely, flower-like structures (FPDA@Pt) and mesoporous spherical structures (MPDA@Pt)—to elucidate their enzyme-mimicking activities, photothermal conversion capabilities, and antitumor efficiencies. The study revealed that, in comparison with MPDA@Pt, FPDA@Pt demonstrated enhanced POD-like activity and superior photothermal conversion efficiency (Fig. 3D and E). This distinction enabled FPDA@Pt to generate ROS and produce heat more efficiently at tumor sites. Notably, the unique flower-like morphology of FPDA@Pt enhanced cellular uptake of the nanozymes, leading to greater accumulation within tumor cells and consequently boosting therapeutic efficacy.

Moreover, when evaluating the performance of nanozymes in treating periodontitis in vivo, it is essential to focus on key characteristics such as their penetration into target periodontal tissues, local concentration, particle size, and cellular uptake efficiency. Generally, smaller-sized nanoparticles have higher tissue penetration capabilities, allowing them to penetrate inflamed areas and reach deeper lesions [56]. However, the size reduction is often accompanied by a shortened half-life in the bloodstream and an accelerated clearance rate from the body, which limits the duration of action in the affected areas [57]. The dual requirement for particle size has driven the creation of multi-stage, size-adjustable systems. These include methods like selectively stripping the outer layers of nanoparticles, surface modification, and cloaking techniques to enhance overall performance. Prussian blue nanoparticles (PB NPs), as a representative multifunctional nanozyme, exhibit substantial advantages in the treatment of periodontitis due to their SOD-like, CAT-like, and POD-like activities [58]. These nanozymes can efficiently scavenge ROS in the affected areas, thereby alleviating oxidative stress and inhibiting inflammatory responses. To delve deeper into the correlation between the physicochemical attributes (e.g., particle size and crystallinity) of PB NPs and their enzyme-like activities, He et al. [59] explored the synthesis of a range of PB NPs by modulating the pH and the concentration of cysteine (Cys) as a stabilizing agent. This approach aimed to optimize the enzyme activity of PB NPs. The study found that as the particle size of PB NPs decreased, their crystallinity decreased and the number of defects increased. These small-sized, low-crystallinity Cys-PB NPs exhibited higher POD-like activity, effectively alleviating in vitro inflammatory responses and scavenging intracellular ROS (Fig. 3F to H).

### Surface modification

Through surface modification techniques, nanozymes can introduce specific chemical groups or functional molecules, thereby optimizing multiple properties. Specifically, these modifications can not only boost their catalytic efficiency and reaction specificity but also enhance the structural robustness and biocompatibility of nanozymes, thereby establishing a solid basis for their deployment in intricate biological settings [60]. This strategy provides a powerful tool for the functional regulation of nanozymes, enabling the customized design of their properties according to specific biomedical needs. In the treatment of periodontitis, surface-modified nanozymes can not only enhance their ability to scavenge ROS by optimizing the exposure of active sites but also improve their targeting and retention at inflamed sites through functional modifications. Additionally, surface modifications can enhance the biocompatibility of nanozymes by regulating their hydrophilicity or reducing protein adsorption, thereby reducing potential side effects in healthy tissues. Han et al. [61] synthesized 3 types of ultrasmall MnFe_2_O_4_ nanozymes with different surface charges, namely, MnFe_2_O_4_-COOH, MnFe_2_O_4_-PEG (polyethylene glycol), and MnFe_2_O_4_-NH_2_, and evaluated their antibacterial efficacy. Owing to their remarkable POD-like activity and diminutive size, these nanozymes exhibited notable antibacterial and anti-biofilm capabilities. They successfully infiltrated biofilms and made contact with bacterial cells. Additionally, the MnFe_2_O_4_ nanozymes performed well in promoting wound healing, tissue repair, and regeneration while also reducing inflammatory responses and effectively accelerating the wound-healing process within 12 d. Specifically, the MnFe_2_O_4_-COOH nanozymes exhibited notable antibacterial efficacy against Gram-positive bacteria, because of their interaction with phosphatidylglycerol (PG) and cardiolipin (CL) on the bacterial membrane, achieving an 80% bacterial removal rate for methicillin-resistant *S. aureus* (*MRSA*). The MnFe_2_O_4_-NH_2_ nanozymes, through interaction with the negatively charged bacterial surface, exhibited the most notable wide-range antibacterial efficacy, with removal rates of 95% for *MRSA* and 85% for *Pseudomonas aeruginosa*. The MnFe_2_O_4_-PEG nanozymes were able to dissipate the membrane potential and reduce adenosine triphosphate (ATP) levels in *MRSA* and *P. aeruginosa*, showing relatively broad-spectrum antibacterial activity.

However, how to perform simple surface modification on nanozymes to achieve targeted delivery while maintaining their catalytic activity remains a challenge. Meng et al. [62] designed a diblock DNA sequence for adsorption onto the surface of CuO, achieving stable circulation in vivo, passive accumulation in tumor tissues, and specific recognition of tumor cells. This approach achieved marked nanocatalytic tumor suppression in a mouse xenograft tumor model without significant cytotoxicity. This work not only provides a new approach for the rational design of DNA-modified nanozymes for catalytic tumor therapy but also offers new insights into the bio-interfacial chemistry between CuO and DNA. Through this strategy, we can overcome the challenge of surface modification of nanozymes while maintaining their catalytic activity, providing a new targeted delivery method for cancer treatment (Fig. 3I). In addition, the surface properties of nanozymes can be adjusted through the coating of polymer layers. To investigate the effect of polymer coatings on catalytic activity, Baldim et al. [63] combined 7.8-nm cerium oxide cores with 6 different copolymers, including 2 types of sodium polyacrylate (SPAA) with different terminal functional groups and 4 types of PEG with various terminal functional groups, successfully preparing 6 different polymer-coated cerium oxide nanoparticles. Studies have shown that polymer coatings do not affect the SOD-like, CAT-like, and OXD-like activities of cerium oxide nanoparticles but unexpectedly enhance their POD-like activity (Fig. 3J). Experiments also found that, compared with nanoparticles coated with SPAA copolymers, those coated with PEG-grafted copolymers performed better in mimicking redox enzyme activities, confirming the advantages of phosphonic acid as an anchoring group on the particle surface. This finding not only surprisingly boosts the POD-like activity of cerium oxide nanoparticles but also highlights the importance of using phosphonic acid groups for surface anchoring of nanoparticles. By employing these sophisticated surface modification strategies, nanozymes have demonstrated extensive promise in the targeted treatment of periodontitis, offering novel approaches to simultaneously address inflammation and promote tissue regeneration.

## The Classification of Nanozymes

The classification of nanozymes is closely related to their applications in the treatment of periodontitis. Different types of nanozymes play unique and complementary roles in various therapeutic aspects of periodontitis due to their differences in catalytic activity stability and functional mechanisms. By precisely controlling the properties of nanozymes, their catalytic efficiency can be optimized in the pathological environment to meet different therapeutic needs, thereby achieving more precise treatment outcomes (Table 1).

**Table.** The application of different types of nanozymes in periodontitis treatment

Type of nanozymes	Nanozymes	Mechanism	Outcome	Reference
Copper-based nanozymes	Cu_2_O@RuO_2_	Promotes angiogenesis Stimulates periodontal bone regeneration	Both in vitro and in vivo experiments showed that Cu_2_O@RuO_2_ substantially alleviated periodontitis symptoms and promoted alveolar tissue regeneration.	[120]
Cerium-based nanozymes	Au@CeO_2_-DMF	Eliminate ROS Immune regulation	In rat models, topical application of the material was able to reduce ROS-induced tissue damage and restore periodontal tissue homeostasis substantially.	[121]
	NAC-PEG@CeO_2_	Antibacterial Immune regulation Eliminate ROS	NA-nanoclusters substantially enhanced macrophage autophagy, leading to notable improvement in periodontitis treatment.	[122]
	Core@shell GNRs@CeO Janus	Antibacterial	The material can produce large amounts of ROS, effectively destroying periodontal pathogens.	[123]
Diamond-based nanozymes	O-NDs	Antibacterial Promotes periodontal tissue restoration	It substantially improves the efficiency of bacterial cell membrane destruction and biofilm decomposition, and accelerates the healing process of periodontal wounds.	[124]
Nanozyme composite materials	Cu-NDs/IL gel	Antibacterial Eliminate ROS Promote permeability	It has an excellent free radical scavenging effect and effectively reduces periodontal inflammation.	[54]

### Metal-based nanozymes

#### Iron-based nanozymes

Iron-based nanozymes are a class of enzyme-mimicking nanomaterials with iron as the active center, which have attracted widespread attention due to their multifunctional catalytic properties. These nanozymes exhibit multiple catalytic activities similar to natural enzymes, including POD, SOD, and CAT functions, enabling them to efficiently scavenge H_2_O_2_ and O_2_^−^ in periodontitis. Through this mechanism, iron-based nanozymes can substantially reduce oxidative stress and inflammatory responses, providing a powerful intervention for the treatment of periodontitis [64]. Shen and colleagues [65] have engineered an innovative iron-based nanozyme (FeSN) to effectively eliminate oral biofilms and manage periodontitis. FeSN is composed of histidine-doped FeS_2_ self-assembly and exhibits extremely high POD-like activity. Antibacterial experiments showed that FeSN exhibited potent activity against *F. nucleatum* when combined with H_2_O_2_, substantially decreasing intracellular glutathione reductase and ATP levels while boosting the concentration of OXD cofactors. Additionally, FeSN has excellent biocompatibility and low cytotoxicity, and it demonstrated marked therapeutic efficacy in a rat model of periodontitis, reducing biofilm formation, inflammation, and alveolar bone loss.

#### Copper-based nanozymes

The catalytic properties of copper-based nanozymes originate from the unique role of copper as the active center and the high specific surface area provided by their nanostructures. These characteristics endow copper-based nanozymes with multiple enzyme-like functions, including SOD, CAT, and POD activities. As a result, copper-based nanozymes have shown extensive application potential in the treatment of periodontitis and provide important technical support for the optimization of related therapeutic strategies.

Recently, photosensitive CuS nanoparticles have garnered attention in the field of bio-nanomedicine due to their intrinsic metal ion biological activity and excellent photothermal response properties [66]. Despite these advantages, CuS nanoparticles still face several challenges in practical applications, such as poor water solubility, a tendency to aggregate, and low production of ROS, which limit their further application in the treatment of periodontitis. To address these issues, researchers have proposed a method of synthesizing CuS nanozymes assisted by biomacromolecules. Utilizing silk fibroin (SF) as a biological scaffold and stabilizing agent, CuS nanozymes were synthesized via in situ growth and subsequently incorporated into a methacrylate hyaluronic acid (HAMA)-based matrix to fabricate the microneedle (MN) patch, namely, the MN-SF/CuS patch [67]. The SF/CuS nanozymes demonstrate a pronounced absorption peak in the near-infrared (NIR) range, along with excellent photothermal stability and efficient conversion performance. Therefore, the MN-SF/CuS patch can rapidly heat up under NIR irradiation, with the temperature controllable by the irradiation intensity, thereby enabling the controllable generation of ROS deep within tissues to kill bacteria. Moreover, the SF/CuS nanozymes show good biocompatibility with gingival fibroblasts (GFs) and cause no marked cellular damage under NIR irradiation. Finally, the MN-SF/CuS patch was proven to be effective in treating periodontitis in a canine periodontitis model, promoting alveolar bone repair and exhibiting good antibacterial and anti-inflammatory activities. Photothermal therapy (PTT) is considered a promising sterilization method with low toxicity and minimal damage to surrounding normal tissues. It also allows precise control over the timing, intensity, and location of light exposure, thereby achieving spatiotemporal control of the therapeutic process.

#### Cerium-based nanozymes

Cerium dioxide (CeO_2_), as a rare earth oxide that has garnered attention in the catalysis field, exhibits excellent SOD-like activity and POD-like activity due to its unique redox ability—rapid conversion between Ce^4+^ and Ce^3+^—and the presence of oxygen vacancies [68]. Combining the properties of CeO_2_ with the treatment of periodontitis can envision an innovative therapeutic strategy. Additionally, the biocompatibility and low toxicity of CeO_2_ are also notable advantages in its biomedical applications. This means that, under the premise of reasonable control of dosage and form, CeO_2_ has the potential to become a safe and effective adjuvant treatment for periodontitis. Undoubtedly, the exploration in this domain remains at an early stage, and further rigorous scientific investigations and clinical trials are anticipated to elucidate the precise therapeutic efficacy and safety profile of CeO_2_ for periodontitis treatment. Yu et al. [69] synthesized CeO_2_ NPs that exhibit SOD-like and CAT-like activities, as well as the ability to scavenge ·OH. In vitro experiments confirmed that CeO_2_ NPs can reduce intracellular ROS levels, exhibit anti-inflammatory and antioxidant activities, and inhibit the mitogen-activated protein kinase (MAPK)–nuclear factor κB (NF-κB) signaling pathway. In  vivo experiments, CeO_2_ NPs inhibited lipopolysaccharide (LPS)-induced local ROS production in the gums, reduced periodontal tissue destruction in a rat ligature-induced periodontitis model, and suppressed bone loss and inflammatory responses. The study indicates that CeO_2_ NPs hold great potential for clinical treatment of periodontitis.

### Single-atom nanozymes

Single-atom nanozymes (SAzymes) exhibit extremely high catalytic activity due to their excellent atomic utilization efficiency, showing extensive potential for application in the treatment of periodontitis [70,71]. By precisely controlling the coordination environment and electronic configuration of the single-atom center, the catalytic mechanism and activity can be finely tuned, thereby effectively intervening in the biomolecules associated with periodontitis. This characteristic not only provides a new theoretical framework and strategy for the treatment of periodontitis but also has the potential to bring about more efficient and safer therapeutic solutions. Inspired by natural enzymes, Liu et al. [72] proposed a rational strategy to effectively regulate the local coordination environment of the planar Fe-N_4_ motif by incorporating spatially axial boron (B) ligands (Fe-B/N-C SAzymes). The tuning effect of the axial B ligand on the electronic structure of the planar Fe-N_4_ active center leads to a notable enhancement in OXD-like, POD-like, and CAT-like activities. Kinetic enzymatic analysis revealed that the catalytic efficiency (*k*_cat_/*K*_M_) of the POD- and CAT-like activities of Fe-B/N-C SAzymes is 6.03 and 2.91 times higher than that of the planar Fe-N_4_ (Fe-N-C SAzymes), respectively. Density functional theory (DFT) calculations suggest that the introduction of spatially axial B ligands alters the charge distribution of the Fe-N_4_ structure. This electronic modulation facilitates the heterolytic cleavage of H_2_O_2_, leading to the rapid formation of ROS intermediates.

Moreover, it optimizes the adsorption–desorption of oxygen intermediates, substantially lowering the energy barrier by promoting the desorption of O_2_. Fe-B/N-C SAzymes can catalyze the conversion of H_2_O_2_ to ·OH through POD-like activity. In addition, they promote the decomposition of H_2_O_2_ to O_2_ and continuously catalyze the conversion of O_2_ to toxic O_2_^−^ and singlet oxygen (^1^O_2_) through redox-like activity, thereby catalyzing a cascade reaction. The inherent photothermal effect of Fe-B/N-C enhances multi-enzyme-like activity, resulting in rapid ROS production. This achieves a synergistic effect between chemodynamic therapy (CDT) and PTT, enhancing antibacterial activity against various periodontal pathogens. This study proposes a new method for the systematic design and fine-tuning of the local coordination environment of SAzymes to achieve superior catalytic performance and effective treatment of periodontitis.

### Nanozyme composites

Hydrogels provide a stable microenvironment for nanozymes, effectively preventing their loss in the dynamic oral environment. Their unique porous structure not only facilitates the loading and release of nanozymes but also enables the sustained and slow release of nanozymes in periodontal tissues, thereby achieving long-lasting therapeutic effects [73,74]. Moreover, the porous structure of hydrogels aids in light capture and multiple scattering, endowing them with potential applications in PDT, which can further enhance therapeutic efficacy, especially in the comprehensive treatment of periodontitis [75,76]. Xu et al. [77] developed a TM/BHT/CuTA hydrogel system through the self-assembly of copper tannic acid coordination nanosheets (CuTA NSs) with triglyceride monostearate and 2,6-di-tert-butyl-4-methylphenol (TM/BHT). Compared with single nanozymes, the negatively charged TM/BHT/CuTA hydrogel system demonstrates enhanced accumulation at inflammatory sites. This is primarily due to its ability to bind strongly to positively charged inflammatory regions through electrostatic interactions, thereby minimizing nanozyme loss, reducing the required dosing frequency, and improving the overall practicality of the nanozyme system. The injectable method of administration is convenient and similar to clinical periodontal drug delivery. Moreover, the TM/BHT hydrogel has enzyme-active ester linkages that respond to the enzyme activity at the inflammatory site. Based on this, CuTA nanozymes can be released on demand to respond to the increase in matrix metalloproteinases (MMPs) in periodontitis. The released CuTA nanozymes exhibit broad-spectrum antibacterial activity, which can inhibit bacteria related to periodontal disease. Meanwhile, as a metal-phenolic nanozyme, it can scavenge various ROS by mimicking the cascade process of SOD and CAT, suppressing oxidative stress. The CuTA nanozymes are capable of modulating macrophage polarization from the pro-inflammatory M1 phenotype to the anti-inflammatory M2 phenotype via the nuclear factor erythroid 2-related factor 2 (Nrf2)/NF-κB pathway. This process effectively reduces the levels of pro-inflammatory cytokines, increases the production of anti-inflammatory cytokines, and promotes the expression of osteogenic genes. As a result, it alleviates inflammation and accelerates tissue regeneration in periodontitis. Overall, the multifunctional nanozyme on-demand release platform (TM/BHT/CuTA) represents an innovative and effective strategy for the treatment of periodontitis.

Different types of nanozymes play multiple roles in the treatment of periodontitis. By selecting or designing specific types of nanozymes based on the pathological characteristics of periodontitis (infection, inflammation, oxidative stress, and tissue destruction), it is possible to achieve comprehensive therapeutic effects including antibacterial, anti-inflammatory, antioxidant, and tissue regeneration. This correlation between classification and function provides scientific guidance for the application of nanozymes in periodontitis treatment and helps to promote the development of personalized and precise therapy.

## The Mechanism of Nanozyme Therapy for Periodontitis

The occurrence and progression of periodontitis begin with the gradual accumulation of pathogenic bacteria in subgingival plaque. These pathogens trigger complex inflammatory responses by stimulating the host’s immune defense mechanisms. However, key the driver of inflammation expansion and tissue destruction is the imbalance in the regulation of the immune system, which may ultimately lead to the irreversible loss of the PDL and alveolar bone. This pathological process not only severely threatens oral health but also may have systemic impacts on overall health. Therefore, we propose 3 core therapeutic strategies focusing on antibacterial, anti-inflammatory, and tissue regeneration. These strategies precisely target different stages of periodontitis development to maximize the protection of the structural and functional integrity of periodontal tissues.

As an emerging type of biocatalyst, nanozymes have demonstrated extensive potential for application in all 3 of the aforementioned therapeutic strategies. In terms of antibacterial activity, nanozymes, with their remarkable oxidative stress capabilities, can effectively eliminate harmful bacteria in the oral cavity and thereby alleviate the damage caused by bacteria to periodontal tissues [78]. By reducing the number of pathogenic bacteria, nanozymes help to slow down the inflammatory response of periodontitis, creating favorable conditions for the subsequent steps of immune regulation and tissue regeneration. In terms of anti-inflammation, nanozymes can modulate the host’s immune response, guiding it toward a balanced state [24,79]. By suppressing excessive inflammatory responses, nanozymes help to reduce damage to periodontal tissues and promote their self-repair. Regarding tissue regeneration, nanozymes can enhance the regenerative capacity of the PDL and alveolar bone. By promoting cell proliferation and differentiation, nanozymes contribute to the restoration of the structural integrity and function of periodontal tissues, thereby improving the patient’s oral health status and quality of life [80,81].

### Antibacterial

Periodontal pathogens, such as *P. gingivalis*, use structures like fimbriae and capsules on their surfaces to colonize periodontal tissues and invade host tissue cells. Moreover, they can evade host immune surveillance to survive and grow, thereby establishing and maintaining an inflammatory toxic environment [82,83]. Therefore, the removal of pathogenic microorganisms is an essential part of periodontal treatment. Currently, most clinical treatments still rely on traditional methods, including supragingival scaling, subgingival curettage, and root planning [84,85]. These methods can only provide temporary relief of local inflammation. However, due to the diverse morphology of periodontal pockets and the complex shape of tooth roots, mechanical treatment alone often fails to effectively remove all dental plaque and calculus and cannot completely control the infection within periodontal tissues. Even with the use of antibiotics, the local drug concentration is low, resulting in poor efficacy. Additionally, systemic adverse reactions are considerable, and there is an increased risk of developing microbial resistance [11]. Compared with traditional treatment methods, nanozymes, with their superior antibacterial properties, can effectively address the aforementioned issues. Over the past few years, research on nanozymes in antibacterial applications has developed rapidly, with 2 main antibacterial mechanisms identified: (a) degradation of extracellular DNA (eDNA) to disrupt the integrity of biofilm and (b) generation of ROS to damage bacterial cell walls or cell membranes [86].

**Fig. 4.**
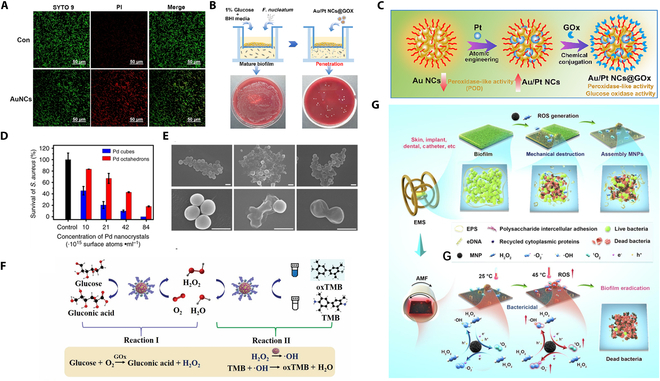
(A) Images of bacterial live/dead fluorescence staining after treatment with AuNCs. Reproduced with permission [90]. Copyright 2022, Springer Nature. (B) Assessment of the ability of Au/Pt NCs@GOx to penetrate the physical barrier of biofilms. (C) Schematic illustration of the synthesis of Au/Pt NCs@GOx with POD and GOx activities. (B and C) Reproduced with permission [91]. Copyright 2023, Elsevier. (D) Survival rate of *S. aureus* after treatment with various concentrations of Pd nanocrystals. (E) Morphological changes on the surface of *S. aureus* cells after exposure to various treatments. (D and E) Reproduced with permission [92]. Copyright 2018, Springer Nature. (F) In the presence of glucose, CoPt@G@GOx with high catalytic activity can catalyze the oxidation of TMB. Reproduced with permission [93]. Copyright 2022, Springer Nature. (G) Illustration of the efficient biofilm removal by MNPs via the electromagnetically actuated magneto-nanozyme-mediated synergetic therapy (EMST) mechanism. Reproduced with permission [94]. Copyright 2022, Elsevier.

Oral biofilms are complex structures composed of various microorganisms and their secreted extracellular polysaccharide matrices, and are important initiators of oral diseases in humans, such as dental caries and periodontitis [87]. *F. nucleatum* plays a vital role in this process. It not only acts as a bridge connecting early and late colonizing microbes, thereby influencing biofilm formation, but also employs virulence factors and metabolic products to damage periodontal tissues. Moreover, it is capable of triggering host immune responses, which in turn may exacerbate periodontal diseases and potentially contribute to the development of systemic conditions [88,89]. Hence, targeting and eliminating *F. nucleatum* can effectively prevent the formation of plaque biofilms and diminish the virulence of dental plaque, which is crucial for preserving oral health and preventing the onset of systemic diseases. Inspired by the intrinsic POD-like catalytic activity of gold nanoclusters (AuNCs), Zhang et al. [90] successfully synthesized ultrasmall AuNCs and investigated their antibacterial activity. The experimental results indicate that AuNCs effectively enhance the antibacterial effect against *F. nucleatum* by increasing the disruption of the bacterial membrane and promoting the production of ROS. Owing to their remarkable penetration capacity, AuNCs are capable of both inhibiting biofilm formation in vitro and disrupting established mature biofilms (Fig. 4A). In a mouse model, we further confirmed the antibacterial efficacy of AuNCs, which reduced biofilm accumulation and improved inflammatory responses. Additionally, we found that after treatment with AuNCs, *F. nucleatum* in the oral cavity could partially repair its cell nucleus. Therefore, AuNCs, as a promising antibacterial material, show great potential in the clinical treatment of dental plaque.

To enhance the antibacterial activity of the material, Wang et al. [91] developed a novel bimetallic nanocluster enzyme (Au/Pt NCs). By substituting Pt atoms, they enhanced the POD-like activity of Au NCs. They engineered a nanocluster enzyme featuring self-enhancing antibacterial properties and superior biocompatibility by conjugating glucose oxidase (GOx) to Au/Pt NCs. This design effectively harnesses the nutrients in the oral environment to transform nontoxic glucose into highly reactive ·OH, which can inhibit and eliminate biofilms caused by *F. nucleatum*. The researchers synthesized the Au/Pt NCs@GOx clusterzyme and characterized its structure and catalytic properties, demonstrating its high efficiency in killing free-floating *F. nucleatum* and disrupting biofilms induced by *F. nucleatum* in vitro experiments (Fig. 4B). Animal experimental results confirmed that the Au/Pt NCs@GOx clusterzyme is safe and can successfully treat periodontitis in rats, reducing inflammation and promoting the regeneration of periodontal tissues. This work represents the first design of a bimetallic nanocluster enzyme (clusterzyme) by incorporating Pt atoms into AuNCs. Studies have shown that compared to Au NCs, the Au/Pt NCs clusterzyme exhibits stronger POD-like activity. The integration of the biocatalyst GOx with Au/Pt NCs (resulting in Au/Pt NCs@GOx) confers cascade catalytic activity to the clusterzyme in the presence of glucose (Fig. 4C). This cascade clusterzyme provides a safe and effective method for treating periodontitis caused by oral biofilms, with potential for clinical application. To investigate the facet-dependent properties of noble metal nanomaterials, researchers have employed a combination of experimental and theoretical approaches to demonstrate that palladium (Pd) nanocrystals display facet-specific OXD and POD-like activities [92]. These activities enable Pd nanocrystals to exhibit antibacterial effects by generating ROS. For Gram-positive bacteria, the antimicrobial efficacy of Pd nanocrystals correlates with their enzyme-like activity. Specifically, crystalline Pd cubes demonstrated higher activity and killed bacteria more effectively than crystalline Pd octahedra (Fig. 4D and E).

However, for Gram-negative bacteria, the opposite trend of antimicrobial activity was observed, with the Pd octahedra showing a greater ability to penetrate the bacterial membrane than the Pd nanocubes, and therefore having a higher antimicrobial activity. These insights enhance our understanding of the facet-dependent enzyme-like activity of noble metal nanomaterials and could propel the development of more effective and targeted antibacterial applications using these materials. Dong et al. [93] developed a novel magnetically driven cascade nanozyme (CoPt@G@GOx) for the treatment of oral biofilms. This nanozyme is composed of GOx and cobalt–platinum–graphene nanocapsules with POD-like activity (Fig. 4F). In the presence of glucose, GOx first oxidizes glucose to produce H_2_O_2_ and gluconic acid. The formation of gluconic acid leads to a decrease in the local microenvironment pH, which in turn enhances the POD-like activity of CoPt@G, enabling it to catalyze the conversion of H_2_O_2_ into highly toxic ·OH. These radicals effectively inhibit bacterial growth. Because H_2_O_2_ is generated catalytically, this approach avoids the potential damage to oral mucosa that could result from high doses of exogenous H_2_O_2_. Meanwhile, the outer graphene protective layer ensures that the metallic core of CoPt@G remains unaffected by the acidic environment, maintaining its stable nanozyme activity. Studies have confirmed that CoPt@G@GOx exhibits highly efficient antibacterial activity under physiological conditions. When further applied to biofilm models, the magnetic driving force substantially increases the retention concentration of CoPt@G@GOx within the biofilm, thereby enhancing its antibacterial activity.

Additionally, the magnetically driven targeting reduces potential damage to normal oral tissues. Iron oxide nanozymes, which exhibit POD-like activity under acidic conditions to convert H_2_O_2_ into highly toxic ROS, still need further improvement in their efficiency for biofilm eradication. Therefore, Ma et al. [94] combined physical and chemical sterilization methods to develop mesoporous iron oxide nanoparticles (MNPs) containing different valence states of iron (0, +2, and +3). These nanoparticles can generate 3 types of ROS in the presence of H_2_O_2_, including ·OH, ^1^O_2_, and O_2_^−^. These ROS not only catalyze bacterial killing but also degrade the extracellular polymeric substances (EPSs) within biofilms. Under the control of an electromagnetic driving system, MNPs can assemble into microclusters, serving a dual function: On one hand, they generate shear forces, acting like a “robotic vacuum cleaner”, to physically disrupt biofilms; on the other hand, under the influence of an alternating magnetic field, the MNP microclusters can produce a hyperthermic effect, which promotes the generation of ROS and thereby enhances the antibacterial efficacy (Fig. 4G). To sum up, this composite nanozyme, characterized by high stability and magnetic responsiveness, offers a straightforward and efficient approach for combating biofilm-associated oral diseases.

### Anti-inflammatory

One of the typical characteristics of periodontitis is the inflammatory destruction of the periodontal supporting tissues. Overactivation of the immune response results in the release of inflammatory mediators, which subsequently impact the PDL, gingiva, cementum, and alveolar bone. This cascade of events can lead to the loss of gingival attachment, increased depth of periodontal pockets, and resorption of the alveolar bone [95]. Although periodontal basic therapy and surgical treatment can control the inflammatory response and promote the regeneration of periodontal tissues to some extent, the therapeutic effects are often limited. If precise intervention could be targeted at the core links in the pathological process, it would be possible to effectively block or slow down the inflammatory response and tissue destruction, thereby achieving better therapeutic outcomes. In this field, nanozymes, as an innovative therapeutic tool, have shown remarkable anti-inflammatory potential. They mainly function by scavenging excess ROS, mimicking the activities of multiple enzymes, targeting the blockade of inflammatory responses, and promoting macrophage polarization, thereby providing an entirely new pathway and strategy for the clinical treatment of periodontitis. This nanozyme-based therapeutic approach holds the potential to substantially enhance the efficacy of periodontitis treatment and mark a new milestone in anti-inflammatory strategies.

#### Regulate inflammatory cytokines

During the immune response, inflammatory cytokines secreted by immune cells and tissue cells play a crucial regulatory role. These inflammatory factors can accelerate the progression of periodontitis by activating transcription factors or signaling pathways related to inflammation, thereby promoting the destruction of periodontal tissues [96]. In contrast, anti-inflammatory cytokines help maintain the integrity of periodontal tissues and inhibit the progression of periodontitis by reducing the expression levels of pro-inflammatory factors [97]. Recent studies have revealed that nanozymes possess the ability to restore the immune balance of periodontal tissues by regulating the secretion of inflammatory cytokines [98]. Specifically, nanozymes can influence the expression of inflammatory cytokines through diverse mechanisms. On one hand, nanozymes can inhibit the production of pro-inflammatory factors. For example, by modulating the activity of inflammation-related signaling pathways such as NF-κB, they reduce the release of pro-inflammatory factors like TNF-α and IL-1β, thereby alleviating the inflammatory response in periodontal tissues. On the other hand, nanozymes can also promote the expression of anti-inflammatory factors, such as increasing the levels of IL-10 and TGF-β, which further enhances the repair and regeneration functions of periodontal tissues. This finding not only provides new ideas for the treatment of periodontitis but also offers valuable insights for the treatment of other inflammatory diseases. Kumawat et al. [99] synthesized silver (Ag) nanozymes using serine as a precursor and further functionalized their surfaces with isonicotinic acid hydrazide (isoniazid), streptomycin, and phosphomolybdic acid. These modifications were aimed at modulating the enzyme-mimicking properties of the nanozymes and enhancing their biological functions. Following comprehensive physicochemical analysis, the Ag-based functional nanozymes were explored for their POD-like activity and favorable blood compatibility. Assessments of cell viability and proliferation in mouse RAW264.7 macrophages validated the excellent biocompatibility of these Ag nanozymes. Additionally, they exhibited notable anti-inflammatory capabilities by modulating the expression of key inflammatory cytokines such as IL-6, IL-1β, and TNF-α.

#### Immune regulation

In the immune system, macrophages not only are the first line of defense against microbial invasion but also play a central role in regulating immune responses. Macrophages can differentiate into different phenotypes in response to various stimuli. Among them, M1 and M2 macrophages are the 2 major phenotypes, each playing distinct roles in immune responses [100]. M1 macrophages, often referred to as classically activated macrophages, are induced by stimuli like LPS and IFN-γ. These macrophages drive inflammatory reactions by secreting pro-inflammatory cytokines, including IL-6 and TNF-α [101]. These cytokines facilitate the recruitment of additional immune cells to the site of infection or injury, thereby triggering the immune response. Meanwhile, M2 macrophages, which are alternatively activated, can further differentiate into distinct subtypes—namely, M2a, M2b, M2c, and M2d—depending on the stimuli and transcriptional regulation they encounter [102]. M2a macrophages are stimulated by IL-4 and IL-13, and they secrete factors that promote fibrosis, such as TGF-β and insulin-like growth factors, thereby facilitating tissue repair. M2b cells express and secrete large amounts of the anti-inflammatory cytokine IL-10 and low levels of IL-12, representing a functional switch from M1 cells. M2c macrophages are induced by IL-10 and strongly exhibit anti-inflammatory activity through the release of large amounts of IL-10. M2d macrophages, also known as tumor-associated macrophages, are involved in the regulation of the tumor microenvironment. Therefore, M2 macrophages have pro-proliferative and pro-angiogenic effects, promoting tissue repair and wound healing. These research findings indicate that precise regulation of macrophage polarization states can not only effectively modulate inflammatory responses but also promote tissue repair and regeneration. This provides a brand-new theoretical basis and strategic direction for the treatment of inflammatory diseases such as periodontitis.

The production of ROS is a key biological mechanism in the process of macrophages phagocytosing and killing microbes. However, an overabundance of ROS can trigger the polarization of macrophages toward the pro-inflammatory M1 phenotype, which in turn amplifies the inflammatory response. Therefore, Wang et al. [103] constructed a nanocomposite named CeO_2_@QU, which combines nanozymes with ROS scavenging functions and natural antioxidants such as flavonoids. In their research, CeO_2_ was initially prepared using a hydrothermal synthesis approach. Following this, the surface of CeO_2_ was aminofunctionalized with 3-aminopropyltriethoxysilane. Quercetin was then added under stirring, ultimately yielding CeO_2_@QU. CeO_2_@QU exhibits an octahedral structure with an average particle size of approximately 120 nm. Experimental results showed that after treatment with CeO_2_@QU, the number of CD86-positive cells decreased (to 40.5%), while the expression of CD206-positive cells significantly increased (to 83.9%). These findings indicate that the CeO_2_@QU nanocomposite has potential applications in regulating macrophage polarization and alleviating inflammatory responses. Animal experimental results showed that after treatment with CeO_2_@QU, the fluorescence intensity of ROS in the animal model was significantly reduced, indicating that the nanocomposite can effectively scavenge excessive ROS. Additionally, the expression of IL-1β, a biomarker of pro-inflammatory M1 macrophages, was down-regulated. These findings confirm that the CeO_2_@QU nanocomposite has the potential to modulate the immune microenvironment. It not only alleviates inflammation by scavenging ROS but also promotes the transformation of M1 macrophages to M2 macrophages, thereby helping to mitigate inflammatory responses and promote tissue repair. This dual-action mechanism makes CeO_2_@QU a promising strategy for the treatment of periodontitis.

### Tissue regeneration

Periodontitis-induced chronic progressive destruction of periodontal tissues is one of the leading causes of tooth loss in adults. To effectively restore the supporting structures of teeth, reduce the risk of tooth loosening and loss, and promote the regeneration of periodontal tissues, it is crucial to focus on innovative treatments. Nanozymes have shown great promise in this therapeutic area, emerging as a key highlight in clinical applications for periodontal treatment.

Periodontal regeneration refers to the complex physiological process by which the damaged periodontal supporting tissues, including cementum, PDL, and alveolar bone, undergo biological reconstruction to restore their structure and function following periodontitis or other destructive lesions. Unlike simple wound healing, true periodontal regeneration demands not only the filling of new tissues but also the specific reconstruction of the structure and function of various tissues with a high degree of organization and biomechanical integration [104]. During the regeneration process, the formation of cementum is one of the key steps, providing a biological basis for the attachment of PDL fibers. New cementum usually originates from dental papilla mesenchymal cells or cementoblasts, and its differentiation is finely regulated by multiple signaling pathways [105]. The reconstruction of the PDL is particularly complex, as it needs not only to regenerate functional fiber structures but also to present directionally arranged fiber bundles (Sharpey’s fibers) from cementum to alveolar bone to maintain the functional mobility of teeth and stress transmission [106]. This highly ordered fiber arrangement is one of the important indicators to judge whether the tissue functional reconstruction is successful in periodontal regeneration [107]. The regeneration of alveolar bone is usually accompanied by the recruitment and activation of bone formation-related cells (such as osteoblasts) and is regulated by growth factors, cytokines, and mechanical signals in the local microenvironment [108]. The coordinated remodeling process among the 3 determines whether the regenerated tissues can be effectively integrated and maintain long-term stability. Overall, periodontal regeneration is a spatiotemporally coupled process involving multiple cells and factors. It requires the synergistic regeneration of cementum, PDL, and alveolar bone in a suitable microenvironment and the reconstruction of anisotropic fiber arrangements to restore the integrity and function of periodontal tissues.

Nanozymes promote periodontal tissue regeneration through multiple mechanisms. First, they substantially improve the microenvironment of damaged tissues, providing more favorable conditions for cell growth and repair. Second, nanozymes can enhance cell signal transduction, helping to activate and regulate cellular activity within periodontal tissues. Additionally, nanozymes can stimulate the differentiation of stem cells, guiding these cells to transform into specific types of cells needed for periodontal tissues, thereby accelerating the regeneration process. In summary, nanozymes play a crucial role in promoting periodontal tissue regeneration. By optimizing the local microenvironment, regulating cell signaling pathways, and inducing the directed differentiation of stem cells, they demonstrate innovative potential in periodontitis treatment, offering unprecedented therapeutic strategies and development prospects in this field.

**Fig. 5.**
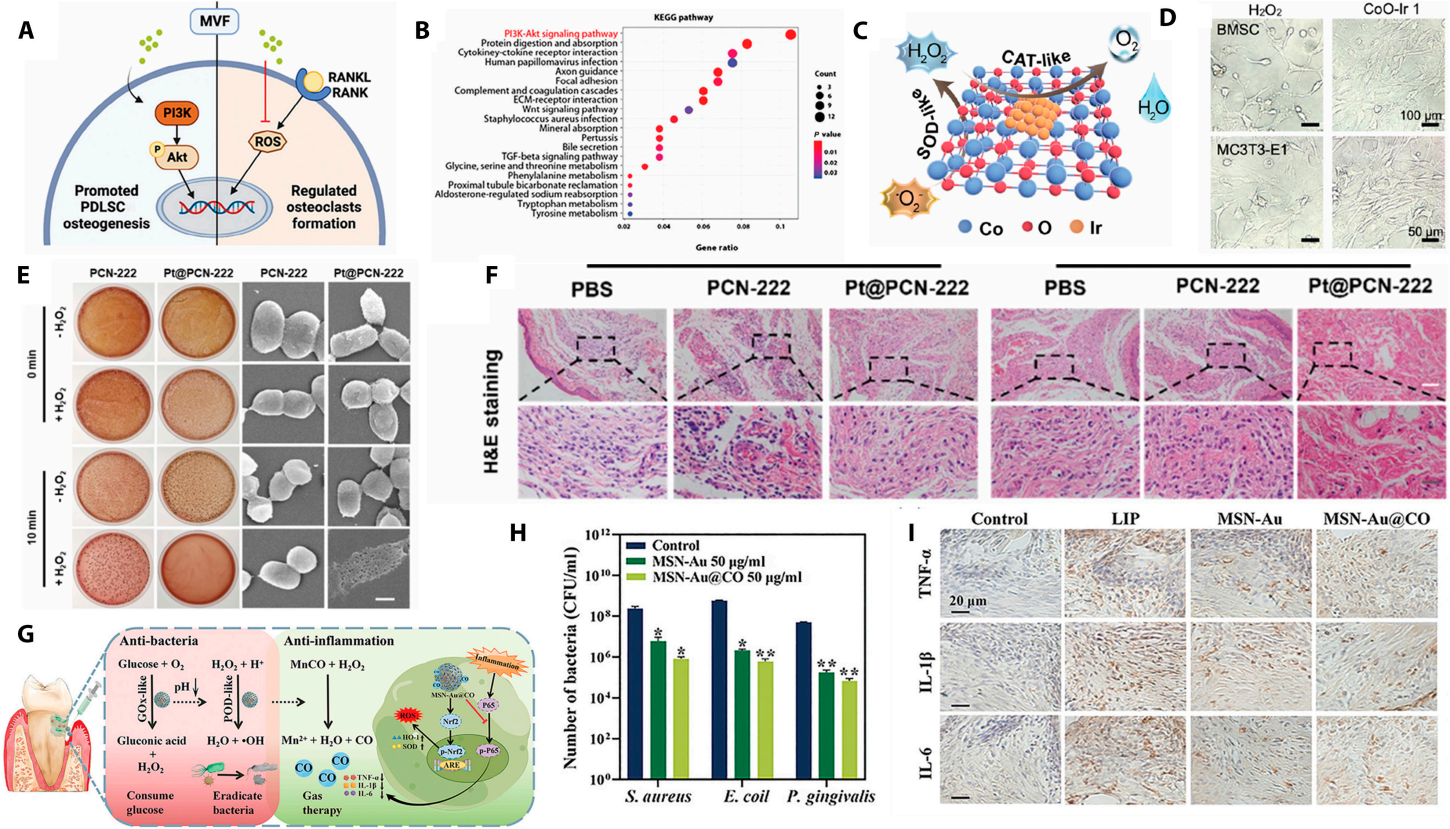
(A) Schematic illustration of the mechanism by which MVF regulates bone remodeling. (B) Kyoto Encyclopedia of Genes and Genomes (KEGG) pathway enrichment analysis of PDLSCs treated with MVF. (A and B) Reproduced with permission [110]. Copyright 2024, John Wiley and Sons. (C) Schematic illustration of the cascade SOD-CAT catalytic activity of CoO−Ir. (D) Images of BMSCs and MC3T3-E1 cells after different treatments. (C and D) Reproduced with permission [112]. Copyright 2023, ACS Publications. (E) Left: Images of bacterial colonization on brain heart infusion (BHI) agar plates after different treatments. Right: Scanning electron microscopy images of *P. gingivalis* after different treatments. (F) Microscopic images of H&E staining of periodontal tissues after different treatments. (E and F) Reproduced with permission [113]. Copyright 2024, Elsevier. (G) Schematic illustration of the antibacterial and anti-inflammatory roles of MSN-Au@CO in the treatment of diabetic periodontitis. (H) Quantities of *S. aureus*, *E. coli*, and *P. gingivalis* under different treatments. (I) Immunohistochemical staining images of TNF-α, IL-10, and IL-6 in gingival tissues after different treatments. (G to I) Reproduced with permission [115]. Copyright 2024, John Wiley and Sons.

PDL stem cells (PDLSCs), as a subpopulation of mesenchymal stem cells, not only possess the characteristics of self-renewal and immune regulation but also have the ability to repair damaged periodontal tissues specifically [109]. These stem cells can differentiate into fibroblasts, osteoblast-like cells, and odontoclast-like cells, and they can generate connective tissue and tooth-like bone tissue with the help of nanomedicine technology. So, controlling the differentiation of PDLSCs has become a promising strategy to promote periodontal tissue repair. This strategy leverages the multipotency of PDLSCs, offering a new direction for the treatment of periodontitis and periodontal tissue engineering. Zhu et al. [110] successfully synthesized the MIL-47(V)-F (MVF) nanozyme, a novel material that mimics the function of glutathione peroxidase (GPx). It can specifically eliminate the key ROS-H_2_O, thereby providing a strategy to appropriately regulate ROS. This strategy helps to limit inflammation, modulate the immune microenvironment, and promote periodontal regeneration. MVF not only functions by eliminating H_2_O_2_ but also directly stimulates the osteogenic differentiation of periodontal stem cells, further promoting regeneration. This effect is partly attributed to the vanadium element in MVF, which plays a crucial role in modulating cellular behavior and enhancing tissue repair. Mechanistically, MVF modulates ROS levels by activating the Nrf2/HO-1 pathway and directly enhances osteogenic differentiation via the phosphatidylinositol 3-kinase (PI3K)/Akt signaling pathway (Fig. 5A and B). In the in vivo experiments, the periodontitis model was established by ligating the maxillary second molars of rats with orthodontic ligature wires. When the ligature wires were placed between the molars, they typically led to plaque accumulation and gingival tissue damage. Micro-computed tomography results revealed that rats treated with MVF exhibited significant improvements in alveolar bone parameters. Moreover, hematoxylin and eosin (H&E) and Masson’s trichrome staining further confirmed that MVF treatment effectively preserved the junctional epithelial attachment to the alveolar bone and the integrity of periodontal tissues. This indicates that MVF not only is involved in the repair of alveolar bone but also has the capacity to regenerate epithelial and fibrous tissues (Fig. S1A to C). Therefore, by leveraging this GPx-mimicking nanozyme and its triple actions of antioxidant, immune modulation, and bone remodeling regulation, a promising therapeutic strategy for periodontitis has been proposed. This strategy positions nanozymes as a powerful asset for advancing precision medicine and provides a novel avenue for periodontitis treatment.

Bone marrow mesenchymal stem cells (BMSCs) are multipotent stem cells that can differentiate into various cell lineages, such as osteoblasts, chondrocytes, adipocytes, myocytes, and more, under specific conditions [111]. In addition, MC3T3-E1 cells are multipotent and capable of differentiating into osteoblasts and osteocytes. They have also been demonstrated to form calcified bone tissue in vitro. This endows both BMSCs and MC3T3-E1 cells with extensive potential for application in periodontal tissue regeneration. Xie et al. [112] developed a novel artificial antioxidant enzyme, namely, cobalt oxide-supported iridium (CoO-Ir), aiming to alleviate local tissue inflammation and bone resorption in periodontitis. The material is characterized by the uniform dispersion of iridium (Ir) nanoclusters on the cobalt oxide (CoO) lattice, forming a stable chemical coupling and enabling strong charge transfer from Co to Ir sites. This unique structure endows CoO-Ir with exceptional catalytic properties, manifested as cascaded and ultrafast SOD- and CAT-like activities (Fig. 5C). Specifically, CoO-Ir exhibits significantly enhanced maximum reaction rates (*V*_max_, 76.249 mg l^−1^ min^−1^) and turnover frequencies (2.736 s^-1^) in the elimination of H₂O₂, both of which surpass those of most reported artificial enzymes. CoO-Ir demonstrates excellent biocompatibility, particularly in proliferation assays of BMSCs and MC3T3-E1, confirming its lack of notable toxicity at safe concentrations. Further experiments in oxidative stress models indicate that CoO-Ir can effectively scavenge intracellular ROS, thereby protecting cells from oxidative damage (Fig. 5D). Thus, CoO-Ir not only performs well in terms of biocompatibility but also has the potential to neutralize oxidative stress in inflammatory microenvironments and restore cellular physiological functions, supporting its application in the medical field. The study reveals that CoO-Ir can effectively clear ROS from BMSCs and MC3T3-E1 cells, protecting them from oxidative stress-induced damage and death. Moreover, CoO-Ir exhibits the ability to promote the differentiation of BMSCs and MC3T3-E1 cells into osteoblasts, showing high osteogenic activity. Specifically, cells treated with CoO-Ir not only display typical osteoblastic morphology but also show significant increases in the expression of early osteogenic marker alkaline phosphatase (ALP), as well as in the levels of key osteogenic proteins BMP-2 and Runx2. In a rat periodontitis model, it was found that CoO-Ir can effectively clear excessive intracellular ROS, thereby protecting periodontal tissues from oxidative damage. After treatment with CoO-Ir, alveolar bone height was restored, and the volume and thickness of trabecular bone increased, indicating that CoO-Ir has osteoinductive activity in promoting bone regeneration (Fig. S1D and E). These findings confirm the therapeutic potential of CoO-Ir in alleviating local tissue inflammation and bone resorption in periodontitis.

### Dual-targeted therapy

As a disease triggered by the interplay of multiple factors, the treatment complexity of periodontitis is becoming increasingly evident. Traditional monotherapy strategies, such as single antimicrobial or immune-regulatory approaches, have shown notable limitations in curbing disease progression. This has spurred researchers to continuously explore more advanced therapeutic means. Against this backdrop, nanotechnology-driven synergistic therapeutic strategies have emerged, especially nanozymes, which are nanomaterials with unique enzyme-like catalytic activities. Their excellent biocompatibility and catalytic performance have endowed them with unprecedented potential in the treatment of periodontitis. Nanozymes can not only efficiently eliminate periodontal pathogens but also precisely modulate the host immune response to mitigate pathological damage to periodontal tissues. Based on this principle, a dual-mode synergistic therapy has been developed as an innovative treatment strategy. By precisely regulating the catalytic activity of nanozymes, researchers can simultaneously achieve the clearance of periodontal pathogens and the optimization of the host immune response, thereby effectively promoting the repair and regeneration of periodontal tissues. In summary, this multidimensional and multi-strategy approach holds the promise of bringing about a revolutionary breakthrough in the treatment of periodontitis.

Researchers have utilized nanomaterials in combination with PDT to treat bacterial infections, including periodontal infections, due to its high efficiency, simplicity, and low propensity for inducing antibiotic resistance. However, the therapeutic efficiency may be limited by the accumulation of endogenous H_2_O_2_ and insufficient O_2_ supply. To address these issues, Dong et al. [113] developed a novel nanomaterial—platinum-doped mesoporous zirconium porphyrin carboxylate metal-organic framework (MOF PCN-222), abbreviated as Pt@PCN-222. This material has the ability to reshape the microenvironment that is unfavorable for the recovery of infected tissues and promotes the restoration of tissues after periodontal disease. Pt@PCN-222 effectively alleviates anaerobic bacterial infections in periodontal tissues by scavenging excess endogenous H_2_O_2_ and generating dissolved oxygen (Fig. 5E). It not only efficiently eradicates bacteria, but in vivo studies using a mouse model of lower incisor periodontal anaerobic bacterial infection further demonstrated that Pt@PCN-222 can significantly suppress the expression of inflammatory cytokines (such as IL-1β, IL-6, and TNF-α) and facilitate the repair of infected tissues (Fig. 5F). After the modeling process, the gingival and vestibular sulcus soft tissues of the lower anterior teeth in all rats appeared dark red, with a soft texture and notable swelling. Following treatment, rats that received the combined treatment of Pt@PCN-222 and light irradiation (L) exhibited a marked reduction in the swelling of the gingival mucosa and vestibular sulcus. The free gingiva became thinner, positioned closer to the cervical area of the lower anterior teeth, and turned pink, indicating a significant decrease in the degree of soft tissue inflammation (Fig. S1F). This study provides a new therapeutic strategy for the long-standing issue of anaerobic bacterial infections in periodontal tissues that has troubled clinical practice and offers an innovative approach to improving treatment methods.

In addition to its antibacterial efficacy, Li et al. [114] developed a novel nanoparticle, ZIF-8:Ce, by doping Ce into zeolite imidazolate framework-8 (ZIF-8). This innovation aims to achieve dual antibacterial and anti-inflammatory effects for the treatment of periodontitis. The research results indicate that a Ce doping ratio of 1% to 10% does not disrupt the regular and uniform structure of ZIF-8. At concentrations below 30 μg/ml, ZIF-8:Ce can continuously release Zn^2+^ and Ce^3+^/Ce^4+^, exhibiting good SOD/CAT activities without cytotoxicity. ZIF-8:Ce demonstrates excellent anti-biofilm properties against periodontal pathogens. Even with a 10% Ce doping level, where the antibacterial effect is slightly reduced, the reduction in colony-forming units (CFUs) still reaches approximately 2 orders of magnitude. Importantly, the anti-inflammatory effect of ZIF-8:Ce gradually increases with the increasing amount of Ce doping. Specifically, ZIF-8:Ce10% more effectively inhibits the expression of pro-inflammatory factors by suppressing the translocation of the NF-κB/p65 subunit (*P* < 0.05). Moreover, ZIF-8:Ce10% drives macrophage polarization toward the M2 phenotype and enhances the secretion of anti-inflammatory cytokines. Therefore, ZIF-8:Ce nanoparticles offer a new approach to developing an effective anti-inflammatory and antibacterial platform for the treatment of periodontitis.

Diabetic periodontitis poses substantial challenges in treatment, characterized by local inflammation, including high blood glucose, bacterial infection, and high oxidative stress. To address this challenge, Wang et al. [115] developed a multi-enzyme synergistic hybrid nanoplatform by decorating Au NPs onto mesoporous silica nanoparticles (MSNs) and loading manganese carbonyl to generate carbon monoxide (CO), thereby constructing the MSN-Au@CO nanoplatform. Au NPs can mimic GOx activity, catalyzing glucose oxidation into H_2_O_2_ and gluconic acid (Fig. 5G). Subsequently, by mimicking POD activity, H_2_O_2_ is converted into ·OH to eliminate bacteria (Fig. 5H). Meanwhile, the generated CO responds to H_2_O_2_ and synergizes with Au NPs to exert anti-inflammatory effects in LPS-challenged macrophages. The potential mechanism of this synergy may involve the induction of Nrf2 to reduce ROS and the inhibition of NF-κB signaling to reduce inflammatory responses. Importantly, the antibacterial and anti-inflammatory effects of MSN-Au@CO were validated in a ligature-induced diabetic rat periodontitis model. Compared with the control group, rats treated with LPS exhibited increased infiltration of immune cells between M2 and M3, disorganized PDL fibers, and enhanced degradation. However, in the groups treated with MSN-Au and MSN-Au@CO, minimal immune cell infiltration was observed, and the PDL fibers were densely and orderly arranged (Fig. S1G and H). In line with the in vitro anti-inflammatory activities of MSN-Au and MSN-Au@CO, immunohistochemical (IHC) staining revealed that these materials notably decreased the levels of inflammatory cytokines in gingival tissues between M2 and M3, as compared to the LPS-treated group (Fig. 5I). Therefore, MSN-Au@CO utilizes glucose-activated cascade reactions to eliminate bacteria and synergistic gas therapy to modulate the immune microenvironment, providing a potential new direction for the treatment of diabetic periodontitis.

Through biomimetic designs such as cell membrane coating, biomimetic nanozymes can actively target sites of inflammation or disease, reduce nonspecific interactions, and minimize the impact on normal tissues while maintaining good biocompatibility [116]. Therefore, Li et al. [117] have developed a functionalized manganese dioxide (MnO_2_) nanoplatform camouflaged with PDLSC membrane (MnO_2_@hPM) for targeting and reprogramming the inflammatory microenvironment in periodontitis. The affinity biomolecules on the PDLSC membrane and the functionalized proteins induced by hypoxic education endow MnO_2_@hPM with the ability to actively target PDLSCs in the inflammatory environment, neutralize various pro-inflammatory factors, and scavenge excessive ROS. The synergistic effects of inflammation suppression and ROS elimination alleviate mitochondrial dysfunction, improve metabolic disorders, and restore the osteogenic potential of inflammation-damaged PDLSCs. In in vivo experiments, MnO_2_@hPM effectively accumulates at periodontitis sites, significantly alleviates periodontitis symptoms, and reduces bone loss in experimental periodontitis, demonstrating optimized tissue regeneration therapeutic performance. This multifunctional biomimetic nanozyme, with good biocompatibility, not only has the ability to target inflammation but also shows synergistic benefits against periodontitis, highlighting its potential as an advanced therapy for other chronic inflammatory diseases.

MOFs have been proven to be materials with the potential to act as nanozymes for scavenging ROS. However, in the context of chronic inflammation, MOFs are unable to directly regulate cellular processes to prevent damaged mitochondria from further generating ROS, which limits their efficacy in treating periodontitis [118]. In this study, Zhu et al. [119] successfully synthesized MnO_2_@UiO-66 (Ce) by introducing MnO_2_ into the nanoscale mesoporous UiO-66 type MOF. The coupling of MnO_2_ with Ce clusters within the MOF channels constructs a SOD/CAT cascade catalytic system. Of particular significance is that the incorporation of manganese endows the MOF with bioactivity, enhancing mitochondrial autophagy to facilitate the removal of damaged mitochondria and restore long-term cellular homeostasis. The results indicate that this synergistic antioxidant system, MnO_2_@UiO-66, can restore mitochondrial homeostasis and the osteogenic activity of PDL cells in vitro, and effectively alleviate inflammatory bone resorption in an in vivo ligature-induced periodontitis model. This study may provide a design strategy that combines an efficient cascade catalytic system with the long-term regulation of cellular homeostasis to combat oxidative stress in chronic inflammation.

In summary, the strategy of nanozyme therapy for periodontitis relies on its ingenious multifunctional design, integrating multiple mechanisms of action such as antibacterial, anti-inflammatory, and tissue repair capabilities, thereby addressing the complex pathological processes of periodontitis fundamentally. This precise and highly efficient therapeutic approach not only provides an innovative treatment pathway for the intervention of periodontal diseases but also paves a broad application prospect for future clinical treatments, with great potential for translation and clinical application value.

## Conclusion and Outlook

With the deepening understanding of the pathophysiology of periodontitis and continuous advancements in nanomaterials science, nano-based therapeutic strategies have rapidly evolved. Among them, nanodrug delivery systems, with their exceptional physicochemical properties and precise targeting capabilities, provide an efficient platform for periodontitis treatment. This review systematically summarizes nanozyme-based therapeutic strategies for periodontitis, aiming to offer theoretical support for disease intervention and the innovative design of nanozymes.

Although nanozymes have shown broad prospects in the diagnosis and treatment of diseases, they still have marked limitations in many aspects that urgently need in-depth research and systematic optimization. First, in the catalytic system using H_2_O_2_ as a substrate, nanozymes often rely on a higher concentration of H_2_O_2_ to maintain catalytic activity. However, ROS plays a dual role in biological systems. On one hand, they are crucial in normal physiological processes, such as acting as signaling molecules to regulate cell functions, participating in immune responses, and eliminating pathogenic microorganisms. ROS are also involved in modulating cell signaling pathways, promoting cell proliferation, migration, and differentiation, as well as the formation of the extracellular matrix. However, when the production of ROS exceeds the cell’s antioxidant capacity, oxidative stress occurs, causing damage to cells and tissues. Excessive ROS can damage biomolecules within cells, such as lipids, proteins, and nucleic acids, leading to cellular dysfunction, cell death, and even chronic inflammation and tissue damage. During the wound-healing process, an overabundance of ROS can increase cell apoptosis and facilitate the spread of pathogens, thereby impeding wound healing. Moreover, oxidative stress is associated with the development and progression of various diseases, including cancer, cardiovascular diseases, and neurodegenerative disorders. Therefore, maintaining the balance of ROS is essential for normal physiological functions and disease treatment. Second, in detection applications in complex biological environments such as blood, nanozymes face issues like nonspecific adsorption, plasma protein interference, and decreased enzyme activity stability. These issues not only affect the sensitivity and specificity of these tests but also pose challenges to their reliability in liquid biopsy and precision diagnosis. In addition, most nanozyme materials currently are based on metals or metal oxides, which have poor biodegradability and tend to stay in the body for a long time, causing potential chronic toxicity and tissue accumulation problems, thus limiting their clinical translation process.

In terms of clinical translation, the bulk synthesis of nanozymes still faces issues such as poor controllability, low reproducibility, and difficulties in standardization. Moreover, their metabolic pathways in vivo and long-term toxicological data are still unclear, preventing them from entering clinical trials on a large scale. Although preliminary studies have shown that some nanozymes have good tissue compatibility and low toxicity in animal models, their long-term safety assessment still lacks systematic validation, especially in the context of chronic diseases or scenarios requiring long-term therapeutic delivery. In addition, although nanozymes have demonstrated excellent catalytic efficiency in vitro experiments, their actual efficiency in complex in vivo microenvironments may change substantially due to local pH, temperature, ionic strength, and other factors, thereby weakening their therapeutic or diagnostic effects. Current research indicates that the catalytic activity of nanozymes is generally lower than that of natural enzymes, which limits their therapeutic efficacy in practical applications. Furthermore, nanozyme catalytic performance is influenced not only by intrinsic factors such as morphology, size, and surface modifications but also by external conditions, including temperature, pH, and substrate concentration. While substantial progress has been made in elucidating how these factors affect nanozyme activity, the specific mechanisms through which the biological microenvironment regulates nanozyme catalysis and its long-term effects remain insufficiently explored. Therefore, developing highly efficient catalytic nanozymes capable of functioning in complex biological systems has become a critical scientific challenge. Therefore, future research should focus on developing nanozyme materials with higher selectivity, lower toxicity, and better degradability and on strengthening the regulation and mechanism elucidation of their catalytic activity in in vivo environments to achieve effective clinical translation.

Meanwhile, interdisciplinary collaboration plays a pivotal role in advancing nanozyme research. The integration of technologies from multiple disciplines not only facilitates innovations in nanozyme synthesis and characterization but also expands their potential applications in medical diagnostics, precision therapy, and environmental management. Such collaborations can reduce research redundancy, enhance experimental efficiency, and accelerate the clinical translation of research findings. More importantly, they enable a multidimensional assessment of nanozyme safety and potential risks, providing a solid scientific foundation for their clinical application. Future research should focus on optimizing nano-based therapeutic strategies to drive breakthrough advancements in nanozyme-based periodontitis treatment, ultimately alleviating patient suffering and reducing the overall burden on healthcare systems.

## Data Availability

Data sharing is not applicable to this article as no datasets were generated or analyzed during the current study.

## References

[1] Heitz-Mayfield LJA. Conventional diagnostic criteria for periodontal diseases (plaque-induced gingivitis and periodontitis). Periodontol 2000. 2024;95(1):10–19. 38831568 10.1111/prd.12579

[2] Zhao C, Wang D, Zhang J, Ge S, Zhan Z, Xu L, Liao S. Associations of social psychological factors and OHRQoL in periodontitis patients: A structural equation modeling study. Patient Prefer Adherence. 2024;18:2359–2372. 39583136 10.2147/PPA.S492070 PMC11585296

[3] Isola G, Santonocito S, Lupi SM, Polizzi A, Sclafani R, Patini R, Marchetti E. Periodontal health and disease in the context of systemic diseases. Mediators Inflamm. 2023;2023:9720947. 37214190 10.1155/2023/9720947 PMC10199803

[4] Carra MC, Rangé H, Caligiuri G, Bouchard P. Periodontitis and atherosclerotic cardiovascular disease: A critical appraisal. Periodontol 2000. 2023;prd.12528. 10.1111/prd.12528 37997210

[5] Marruganti C, Suvan JE, D’Aiuto F. Periodontitis and metabolic diseases (diabetes and obesity): Tackling multimorbidity. Periodontol 2000. 2023;prd.12536. 10.1111/prd.12536 37845800

[6] Walther K, Gröger S, Vogler JAH, Wöstmann B, Meyle J. Inflammation indices in association with periodontitis and cancer. Periodontol 2000. 2024;96(1):281–315. 39317462 10.1111/prd.12612 PMC11579835

[7] Paraguassu EC. Basic periodontitis manual: What it is, symptoms and treatments. Braz J Implantol Health Sci. 2023;5(2):01–03.

[8] Wu J, Lin L, Xiao J, Zhao J, Wang N, Zhao X, Tan B. Efficacy of scaling and root planning with periodontal endoscopy for residual pockets in the treatment of chronic periodontitis: A randomized controlled clinical trial. Clin Oral Invest. 2022;26(1):513–521. 10.1007/s00784-021-04029-w 34145479

[9] Radu C-M, Radu C, Arbănaşi E-M, Hogea T, Murvai V, Chiș I-A, Zaha D. Exploring the efficacy of novel therapeutic strategies for periodontitis: A literature review. Life. 2024;14(4):468. 38672739 10.3390/life14040468 PMC11050937

[10] Song YW, Nam J, Kim J, Lee Y, Choi J, Min HS, Yang H, Cho Y, Hwang S, Son J, et al. Hyaluronic acid-based minocycline-loaded dissolving microneedle: Innovation in local minocycline delivery for periodontitis. Carbohydr Polym. 2025;349(Pt 8): Article 122976. 39638519 10.1016/j.carbpol.2024.122976

[11] de la Fuente-Nunez C, Cesaro A, Hancock REW. Antibiotic failure: Beyond antimicrobial resistance. Drug Resist Updat. 2023;71: Article 101012. 37924726 10.1016/j.drup.2023.101012 PMC12224857

[12] Golub LM, Lee H-M. Periodontal therapeutics: Current host-modulation agents and future directions. Periodontol 2000. 2020;82(1):186–204. 31850625 10.1111/prd.12315 PMC6973248

[13] Hosseini Hooshiar M, Badkoobeh A, Kolahdouz S, Tadayonfard A, Mozaffari A, Nasiri K, Salari S, Safaralizadeh R, Yasamineh S. The potential use of nanozymes as an antibacterial agents in oral infection, periodontitis, and peri-implantitis. J Nanobiotechnology. 2024;22(1):207. 38664778 10.1186/s12951-024-02472-x PMC11044492

[14] Bilal M, Khaliq N, Ashraf M, Hussain N, Baqar Z, Zdarta J, Jesionowski T, Iqbal HMN. Enzyme mimic nanomaterials as nanozymes with catalytic attributes. Colloids Surf B Biointerfaces. 2023;221: Article 112950. 36327773 10.1016/j.colsurfb.2022.112950

[15] Zhang Q, Wang Z, Shen S, Wang J, Cao J, Deng Y, Meng H, Ma L. Integrating enzyme-nanoparticles bring new prospects for the diagnosis and treatment of immune dysregulation in periodontitis. Front Cell Infect Microbiol. 2024;14:1494651. 39554809 10.3389/fcimb.2024.1494651 PMC11564189

[16] Xiong H, Zhao Y, Xu Q, Xie X, Wu J, Hu B, Chen S, Cai X, Zheng Y, Fan C. Biodegradable hollow-structured nanozymes modulate phenotypic polarization of macrophages and relieve hypoxia for treatment of osteoarthritis. Small. 2022;18(32):e2203240. 35843877 10.1002/smll.202203240

[17] Guo D, Liu H, Zhao S, Lu X, Wan H, Zhao Y, Liang X, Zhang A, Wu M, Xiao Z, et al. Synergistic rheumatoid arthritis therapy by interrupting the detrimental feedback loop to orchestrate hypoxia M1 macrophage polarization using an enzyme-catalyzed nanoplatform. Bioact Mater. 2024;41:221–238. 39149592 10.1016/j.bioactmat.2024.07.026 PMC11324459

[18] He H, Han Q, Wang S, Long M, Zhang M, Li Y, Zhang Y, Gu N. Design of a multifunctional nanozyme for resolving the proinflammatory plaque microenvironment and attenuating atherosclerosis. ACS Nano. 2023;17(15):14555–14571. 37350440 10.1021/acsnano.3c01420

[19] Pant G, Singh S, Choudhary PK, Ramamurthy PC, Singh H, Garlapati D, Singh J, Kumar G, Khan NA, Zahmatkesh S. Nanozymes: Advance enzyme-mimicking theragnostic tool: A review. Clean Techn Environ Policy. 2024;26:3685–3695.

[20] Abdulkareem AA, Al-Taweel FB, Al-Sharqi AJB, Gul SS, Sha A, Chapple ILC. Current concepts in the pathogenesis of periodontitis: From symbiosis to dysbiosis. J Oral Microbiol. 2023;15(1):2197779. 37025387 10.1080/20002297.2023.2197779 PMC10071981

[21] Luo W, Du C, Huang H, Kong J, Ge Z, Lin L, Wang H. The role of macrophage death in periodontitis: A review. Inflammation. 2024;47(6):1889–1901. 38691250 10.1007/s10753-024-02015-4

[22] Ding J, Zhao C, Gao L. Metabolism of periodontal pathobionts: Their regulatory roles in the dysbiotic microbiota. Mol Oral Microbiol. 2023;38(3):181–188. 36811357 10.1111/omi.12409

[23] Das A, Patro S, Simnani FZ, Singh D, Sinha A, Kumari K, Rao PV, Singh S, Kaushik NK, Panda PK, et al. Biofilm modifiers: The disparity in paradigm of oral biofilm ecosystem. Biomed Pharmacother. 2023;164: Article 114966. 37269809 10.1016/j.biopha.2023.114966

[24] Peng S, Fu H, Li R, Li H, Wang S, Li B, Sun J. A new direction in periodontitis treatment: Biomaterial-mediated macrophage immunotherapy. J Nanobiotechnol. 2024;22(1):359. 10.1186/s12951-024-02592-4 PMC11193307 38907216

[25] Bassani B, Cucchiara M, Butera A, Kayali O, Chiesa A, Palano MT, Olmeo F, Gallazzi M, Dellavia CPB, Mortara L, et al. Neutrophils’ contribution to periodontitis and periodontitis-associated cardiovascular diseases. Int J Mol Sci. 2023;24(20):15370. 37895050 10.3390/ijms242015370 PMC10607037

[26] Wilensky A, Segev H, Mizraji G, Shaul Y, Capucha T, Shacham M, Hovav AH. Dendritic cells and their role in periodontal disease. Oral Dis. 2014;20(2):119–126. 23656605 10.1111/odi.12122

[27] Kini V, Mohanty I, Telang G, Vyas N. Immunopathogenesis and distinct role of Th17 in periodontitis: A review. J Oral Biosci. 2022;64(2):193–201. 35489583 10.1016/j.job.2022.04.005

[28] Gemmell E, Seymour GJ. Immunoregulatory control of Th1/Th2 cytokine profiles in periodontal disease. Periodontol 2000. 2004;35:21–41. 15107056 10.1111/j.0906-6713.2004.003557.x

[29] Stashenko P, Gonçalves RB, Lipkin B, Ficarelli A, Sasaki H, Campos-Neto A. Th1 immune response promotes severe bone resorption caused by Porphyromonas gingivalis. Am J Pathol. 2007;170(1):203–213. 17200194 10.2353/ajpath.2007.060597 PMC1762702

[30] Junttila IS. Tuning the cytokine responses: An update on interleukin (IL)-4 and IL-13 receptor complexes. Front Immunol. 2018;9:888. 29930549 10.3389/fimmu.2018.00888 PMC6001902

[31] Wang W, Wang X, Lu S, Lv H, Zhao T, Xie G, du Y, Fan Y, Xu L. Metabolic disturbance and Th17/Treg imbalance are associated with progression of gingivitis. Front Immunol. 2021;12: Article 670178. 34234776 10.3389/fimmu.2021.670178 PMC8257051

[32] Bunte K, Beikler T. Th17 cells and the IL-23/IL-17 axis in the pathogenesis of periodontitis and immune-mediated inflammatory diseases. Int J Mol Sci. 2019;20(14):3394. 31295952 10.3390/ijms20143394 PMC6679067

[33] Park H, Li Z, Yang XO, Chang SH, Nurieva R, Wang Y-H, Wang Y, Hood L, Zhu Z, Tian Q, et al. A distinct lineage of CD4 T cells regulates tissue inflammation by producing interleukin 17. Nat Immunol. 2005;6(11):1133–1141. 16200068 10.1038/ni1261 PMC1618871

[34] Deng J, Lu C, Zhao Q, Chen K, Ma S, Li Z. The Th17/Treg cell balance: Crosstalk among the immune system, bone and microbes in periodontitis. J Periodontal Res. 2022;57(2):246–255. 34878170 10.1111/jre.12958

[35] Almubarak A, Tanagala KKK, Papapanou PN, Lalla E, Momen-Heravi F. Disruption of monocyte and macrophage homeostasis in periodontitis. Front Immunol. 2020;11:330. 32210958 10.3389/fimmu.2020.00330 PMC7067288

[36] Mo K, Wang Y, Lu C, Li Z. Insight into the role of macrophages in periodontitis restoration and development. Virulence. 2024;15(1):2427234. 39535076 10.1080/21505594.2024.2427234 PMC11572313

[37] Li J, Li M, Zhang C, Fei Y, Wang Y, Zhong Z, Peng C, Li M, Gui S, Guo J. Active targeting microemulsion-based thermosensitive hydrogel against periodontitis by reconstructing Th17/Treg homeostasis via regulating ROS-macrophages polarization cascade. Int J Pharm. 2024;659: Article 124263. 38815639 10.1016/j.ijpharm.2024.124263

[38] Wang Z, Saxena A, Yan W, Uriarte SM, Siqueira R, Li X. The impact of aging on neutrophil functions and the contribution to periodontitis. Int J Oral Sci. 2025;17(1):10. 39819982 10.1038/s41368-024-00332-w PMC11739572

[39] Yin C, Fu L, Guo S, Liang Y, Shu T, Shao W, Xia H, Xia T, Wang M. Senescent fibroblasts drive FAP/OLN imbalance through mTOR signaling to exacerbate inflammation and bone resorption in periodontitis. Adv Sci. 2024;12(7):2409398. 10.1002/advs.202409398 PMC11831441 39716898

[40] Amato M, Polizzi A, Viglianisi G, Leonforte F, Mascitti M, Isola G. Impact of periodontitis and oral dysbiosis metabolites in the modulation of accelerating ageing and human senescence. Metabolites. 2025;15(1):35. 39852378 10.3390/metabo15010035 PMC11767177

[41] da Silva RCM, da Silva LGC, Martins AA, de Araújo CM, Martins ARLA. Adjunctive photobiomodulation to basic periodontal therapy using different low-power laser application techniques: A systematic review and meta-analysis. Lasers Med Sci. 2024;39(1):207. 39093490 10.1007/s10103-024-04148-2

[42] Shetty B, Divakar DD, Jameel AHA, Almalki SA, Gowdar IM, Dewan H. Effect of non-surgical periodontal therapy with adjunct photodynamic therapy on periodontal and glycemic statuses in prediabetic patients with periodontal disease. Photodiagn Photodyn Ther. 2023;42: Article 103362. 10.1016/j.pdpdt.2023.103362 36841278

[43] Gerzile A, Naziker Y, Özer E, Ertugrul AS. Impact of surgical and non-surgical periodontal therapy on quality of life in case of periodontitis. Oral Dis. 2025. 10.1111/odi.15316 PMC12423493 40106822

[44] Jepsen K, Sculean A, Jepsen S. Complications and treatment errors related to regenerative periodontal surgery. Periodontol 2000. 2023;92(1):120–134. 37435999 10.1111/prd.12504

[45] Fayazi M, Rostami M, Moghaddam MA, Nasiri K, Tadayonfard A, Roudsari MB, Ahmad HM, Parhizgar Z, Yazdi AM. A state-of-the-art review of the recent advances in drug delivery systems for different therapeutic agents in periodontitis. J Drug Target. 2024;33(5):612–647. 39698877 10.1080/1061186X.2024.2445051

[46] Deshwal A, Saxena K, Sharma G, Sheikh FA, Seth CS, Tripathi RM. Nanozymes: A comprehensive review on emerging applications in cancer diagnosis and therapeutics. Int J Biol Macromol. 2024;256(Pt 1): Article 128272. 38000568 10.1016/j.ijbiomac.2023.128272

[47] Li R, Hou X, Li L, Guo J, Jiang W, Shang W. Application of metal-based nanozymes in inflammatory disease: A review. Front Bioeng Biotechnol. 2022;10:920213. 35782497 10.3389/fbioe.2022.920213 PMC9243658

[48] Singh S. Antioxidant nanozymes as next-generation therapeutics to free radical-mediated inflammatory diseases: A comprehensive review. Int J Biol Macromol. 2024;260(Pt 1): Article 129374. 38242389 10.1016/j.ijbiomac.2024.129374

[49] Wu Y, Chen W, Wang C, Xing D. Nanozyme-activating prodrug therapies: A review. Chin Chem Lett. 2024;35(2): Article 109096.

[50] Wei M, Lee J, Xia F, Lin P, Hu X, Li F, Ling D. Chemical design of nanozymes for biomedical applications. Acta Biomater. 2021;126:15–30. 33652165 10.1016/j.actbio.2021.02.036

[51] Lai C-M, Xiao X-S, Chen J-Y, He W-Y, Wang S-S, Qin Y, He SH. Revolutionizing nanozymes: The synthesis, enzyme-mimicking capabilities of carbon dots, and advancements in catalytic mechanisms. Int J Biol Macromol. 2025;293: Article 139284. 39736288 10.1016/j.ijbiomac.2024.139284

[52] Yang M, Liu Y, Zhang L, Qian Y, Li N, Zhang G, Hu Y, Li X, Ge Y, Peng Y, et al. Highly conjugated nanozyme with non coordination saturation for cascaded enhanced POD reaction driving antibacterial therapy. Adv Funct Mater. 2024;34(42):2404894.

[53] Liu C, Zhao X, Wang Z, Zhao Y, Li R, Chen X, Chen H, Wan M, Wang X. Metal-organic framework-modulated Fe_3_O_4_ composite au nanoparticles for antibacterial wound healing via synergistic peroxidase-like nanozymatic catalysis. J Nanobiotechnology. 2023;21(1):427. 37968680 10.1186/s12951-023-02186-6 PMC10647143

[54] Gao Y, Zhang W, Xue R, Shu Y, Wang J. An ionic gel incorporating copper nanodots with antibacterial and antioxidant dual functions for deep tissue penetration treatment of periodontitis in rats. Biomater Sci. 2023;11(10):3547–3560. 37000509 10.1039/d3bm00309d

[55] Xu Z, Jiang J, Li Y, Hu T, Gu J, Zhang P, Fan L, Xi J, Han J, Guo R. Shape-regulated photothermal-catalytic tumor therapy using polydopamine@Pt nanozymes with the elicitation of an immune response. Small. 2024;20(20):2309096. 10.1002/smll.202309096 38054612

[56] Wang Z, Zhang R, Yan X, Fan K. Structure and activity of nanozymes: Inspirations for de novo design of nanozymes. Mater Today. 2020;41:81–119.

[57] Su L, Qin S, Xie Z, Wang L, Khan K, Tareen AK, Li D, Zhang H. Multi-enzyme activity nanozymes for biosensing and disease treatment. Coord Chem Rev. 2022;473: Article 214784.

[58] Zhang W, Hu S, Yin J-J, He W, Lu W, Ma M, Gu N, Zhang Y. Prussian blue nanoparticles as multienzyme mimetics and reactive oxygen species scavengers. J Am Chem Soc. 2016;138(18):5860–5865. 26918394 10.1021/jacs.5b12070

[59] He Y, Bianco A, Ménard-Moyon C. Size and crystallinity effects on enzymatic activity and anti-inflammatory properties of cysteine-assisted Prussian blue nanozymes. J Colloid Interface Sci. 2025;679(Pt A):930–938. 39413589 10.1016/j.jcis.2024.10.008

[60] Hou J, Xianyu Y. Tailoring the surface and composition of nanozymes for enhanced bacterial binding and antibacterial activity. Small. 2023;19(42):2302640. 10.1002/smll.202302640 37322391

[61] Han J, Chen Y, Xiang X, Wang T, Shen J, Zhang N, Liang C, Liu X, Ma X. A comparative analysis of the antibacterial spectrum of ultrasmall manganese ferrite nanozymes with varied surface modifications. ACS Appl Mater Interfaces. 2024;16(12):14385–14404. 38489475 10.1021/acsami.3c16490

[62] Meng Y, Chen Y, Zhu J, Qi Y, Ding J, Zhou W. Polarity control of DNA adsorption enabling the surface functionalization of CuO nanozymes for targeted tumor therapy. Mater Horiz. 2021;8(3):972–986. 34821328 10.1039/d0mh01372b

[63] Baldim V, Nisha Y, Bia N, Graillot A, Loubat C, Singh S, Karakoti AS, Berret JF. Polymer coated cerium oxide nanoparticles as oxidoreductase-like catalysts. arXiv. 2020. 10.48550/arXiv.2008.08492. 32812730

[64] Fu R, Ma Z, Zhao H, Jin H, Tang Y, He T, Ding Y, Zhang J, Ye D. Research progress in iron-based nanozymes: Catalytic mechanisms, classification, and biomedical applications. Anal Chem. 2023;95(29):10844–10858. 37438259 10.1021/acs.analchem.3c01005

[65] Shen B, Yang L, Xu H, Zhang Y, Ming D, Zhu L, Wang Y, Jiang L. Detection and treatment of biofilm-induced periodontitis by histidine-doped FeSN nanozyme with ultra-high peroxidase-like activity. J Colloid Interface Sci. 2023;650(Pt A):211–221. 37402327 10.1016/j.jcis.2023.06.188

[66] Huang J, Zhou J, Zhuang J, Gao H, Huang D, Wang L, Wu W, Li Q, Yang DP, Han MY. Strong near-infrared absorbing and biocompatible CuS nanoparticles for rapid and efficient photothermal ablation of gram-positive and -negative bacteria. ACS Appl Mater Interfaces. 2017;9(42):36606–36614. 28976189 10.1021/acsami.7b11062

[67] Chen H, Yu N, Wang J, Zhang S, Cao L, Zhou M, Xu Z, Lin S, Yin S, Jiang X, et al. Construction of versatile fibroin/nanozyme hybrid microneedles with controllable phototherapeutic sterilization property against periodontitis. Nano Today. 2024;56: Article 102297.

[68] Wang G, Zhang J, He X, Zhang Z, Zhao Y. Ceria nanoparticles as enzyme mimetics. Chin J Chem. 2017;35(6):791–800.

[69] Yu Y, Zhao S, Gu D, Zhu B, Liu H, Wu W, Wu J, Wei H, Miao L. Cerium oxide nanozyme attenuates periodontal bone destruction by inhibiting the ROS–NFκB pathway. Nanoscale. 2022;14(7):2628–2637. 35088792 10.1039/d1nr06043k

[70] Huang L, Chen J, Gan L, Wang J, Dong S. Single-atom nanozymes. Sci Adv. 2019;5(5):eaav5490. 31058221 10.1126/sciadv.aav5490 PMC6499548

[71] Fu Z, Fan K, He X, Wang Q, Yuan J, Lim KS, Tang JN, Xie F, Cui X. Single-atom-based nanoenzyme in tissue repair. ACS Nano. 2024;18(20):12639–12671. 38718193 10.1021/acsnano.4c00308

[72] Liu W, Shi E, Wu H, Liang Y, Chen M, Zhang H, Zhang R, Li X, Wang Y, Zhang L. Spatially axial boron coordinated single-atom nanozymes with boosted multi-enzymatic performances for periodontitis treatment. Adv Funct Mater. 2024;34(39):2403386.

[73] Li Q, Wang D, Xiao C, Wang H, Dong S. Advances in hydrogels for periodontitis treatment. ACS Biomater Sci Eng. 2024;10(5):2742–2761. 38639082 10.1021/acsbiomaterials.4c00220

[74] Xu X, Jerca VV, Hoogenboom R. Bioinspired double network hydrogels: From covalent double network hydrogels *via* hybrid double network hydrogels to physical double network hydrogels. Mater Horiz. 2021;8(4):1173–1188. 34821910 10.1039/d0mh01514h

[75] Xu Y, Chen H, Fang Y, Wu J. Hydrogel combined with phototherapy in wound healing. Adv Healthc Mater. 2022;11(16):2200494. 10.1002/adhm.202200494 35751637

[76] Pan Q, Zong Z, Li H, Xie L, Zhu H, Wu D, Liu R, He B, Pu Y. Hydrogel design and applications for periodontitis therapy: A review. Int J Biol Macromol. 2025;284(Pt 1): Article 137893. 39571840 10.1016/j.ijbiomac.2024.137893

[77] Xu Y, Luo Y, Weng Z, Xu H, Zhang W, Li Q, Liu H, Liu L, Wang Y, Liu X, et al. Microenvironment-responsive metal-phenolic nanozyme release platform with antibacterial, ROS scavenging, and osteogenesis for periodontitis. ACS Nano. 2023;17(19):18732–18746. 37768714 10.1021/acsnano.3c01940

[78] Zhou C, Wang Q, Cao H, Jiang J, Gao L. Nanozybiotics: Advancing antimicrobial strategies through biomimetic mechanisms. Adv Mater. 2024;36(33):2403362. 10.1002/adma.202403362 38874860

[79] Yang B, Pang X, Li Z, Chen Z, Wang Y. Immunomodulation in the treatment of periodontitis: Progress and perspectives. Front Immunol. 2021;12:781378. 34868054 10.3389/fimmu.2021.781378 PMC8640126

[80] Mou X, Wu Q, Zhang Z, Liu Y, Zhang J, Zhang C, Chen X, Fan K, Liu H. Nanozymes for regenerative medicine. Small Methods. 2022;6(11):e2200997. 36202750 10.1002/smtd.202200997

[81] Xiong Y, Mi B, Liu G, Zhao Y. Microenvironment-sensitive nanozymes for tissue regeneration. Biomaterials. 2024;309: Article 122585. 38692147 10.1016/j.biomaterials.2024.122585

[82] Zheng S, Yu S, Fan X, Zhang Y, Sun Y, Lin L, Wang H, Pan Y, Li C. *Porphyromonas gingivalis* survival skills: Immune evasion. J Periodontal Res. 2021;56(6):1007–1018. 34254681 10.1111/jre.12915

[83] Zhou T, Xu W, Wang Q, Jiang C, Li H, Chao Y, Sun Y, A L. The effect of the “oral-gut” axis on periodontitis in inflammatory bowel disease: A review of microbe and immune mechanism associations. Front Cell Infect Microbiol. 2023;13:1132420. 36923589 10.3389/fcimb.2023.1132420 PMC10008960

[84] Albandar JM. Aggressive and acute periodontal diseases. Periodontol 2000. 2014;65(1):7–12. 24738583 10.1111/prd.12013

[85] Paterson M, Johnston W, Sherriff A, Culshaw S. Periodontal instrumentation technique: An exploratory analysis of clinical outcomes and financial aspects. Br Dent J. 2023;1–8. 10.1038/s41415-022-5405-1 PMC9838345 36624308

[86] Yang D, Chen Z, Gao Z, Tammina SK, Yang Y. Nanozymes used for antimicrobials and their applications. Colloids Surf B Biointerfaces. 2020;195: Article 111252. 32679446 10.1016/j.colsurfb.2020.111252

[87] Ray RR, Pattnaik S. Technological advancements for the management of oral biofilm. Biocatal Agric Biotechnol. 2024;56: Article 103017.

[88] Stokowa-Sołtys K, Wojtkowiak K, Jagiełło K. Fusobacterium nucleatum—Friend or foe? J Inorg Biochem. 2021;224: Article 111586. 34425476 10.1016/j.jinorgbio.2021.111586

[89] Pignatelli P, Nuccio F, Piattelli A, Curia MC. The role of *Fusobacterium nucleatum* in oral and colorectal carcinogenesis. Microorganisms. 2023;11(9):2358. 37764202 10.3390/microorganisms11092358 PMC10537357

[90] Zhang Y, Chen R, Wang Y, Wang P, Pu J, Xu X, Chen F, Jiang L, Jiang Q, Yan F. Antibiofilm activity of ultra-small gold nanoclusters against Fusobacterium nucleatum in dental plaque biofilms. J Nanobiotechnology. 2022;20(1):470. 36329432 10.1186/s12951-022-01672-7 PMC9632159

[91] Wang Y, Li C, Shen B, Zhu L, Zhang Y, Jiang L. Ultra-small Au/Pt NCs@GOX clusterzyme for enhancing cascade catalytic antibiofilm effect against *F. nucleatum*-induced periodontitis. Chem Eng J. 2023;466: Article 143292.

[92] Fang G, Li W, Shen X, Perez-Aguilar JM, Chong Y, Gao X, Chai Z, Chen C, Ge C, Zhou R. Differential Pd-nanocrystal facets demonstrate distinct antibacterial activity against Gram-positive and Gram-negative bacteria. Nat Commun. 2018;9(1):129. 29317632 10.1038/s41467-017-02502-3 PMC5760645

[93] Dong Q, Li Z, Xu J, Yuan Q, Chen L, Chen Z. Versatile graphitic nanozymes for magneto actuated cascade reaction-enhanced treatment of *S. mutans* biofilms. Nano Res. 2022;15:9800–9808.

[94] Ma X, Wang L, Wang P, Liu Z, Hao J, Wu J, Chu G, Huang M, Mair LO, Huang C, et al. An electromagnetically actuated magneto-nanozyme mediated synergistic therapy for destruction and eradication of biofilm. Chem Eng J. 2022;431(Pt 1): Article 133971.

[95] Pan W, Wang Q, Chen Q. The cytokine network involved in the host immune response to periodontitis. Int J Oral Sci. 2019;11(3):30. 31685798 10.1038/s41368-019-0064-z PMC6828663

[96] Wang RP-H, Huang J, Chan KWY, Leung WK, Goto T, Ho Y-S, Chang RCC. IL-1β and TNF-α play an important role in modulating the risk of periodontitis and Alzheimer’s disease. J Neuroinflammation. 2023;20(1):71. 36915108 10.1186/s12974-023-02747-4 PMC10012546

[97] Koshy B, Rees JS, Farnell DDJJ, Wei X-Q, Waddington RJ. Array analysis for T-cell associated cytokines in gingival crevicular fluid: Identifying altered profiles associated with periodontal disease status. J Dent. 2019;85:39–46. 31028890 10.1016/j.jdent.2019.04.009

[98] Ju Y, Liu X, Ye X, Dai M, Fang B, Shen X, Liu L. Nanozyme-based remodeling of disease microenvironments for disease prevention and treatment: A review. ACS Appl Nano Mater. 2023;6(15):13792–13823.

[99] Kumawat M, Madhyastha H, Singh M, Jain D, Daima HK. Functional silver nanozymes regulate cell inflammatory cytokines expression in mouse macrophages. Colloids Surf A Physicochem Eng Asp. 2022;650: Article 129294.

[100] Strizova Z, Benesova I, Bartolini R, Novysedlak R, Cecrdlova E, Foley LK, Striz I. M1/M2 macrophages and their overlaps – myth or reality? Clin Sci. 2023;137(15):1067–1093. 10.1042/CS20220531 PMC10407193 37530555

[101] Chen S, Saeed AFUH, Liu Q, Jiang Q, Xu H, Xiao GG, Rao L, Duo Y. Macrophages in immunoregulation and therapeutics. Signal Transduct Target Ther. 2023;8(1):207. 37211559 10.1038/s41392-023-01452-1 PMC10200802

[102] Liu J, Geng X, Hou J, Wu G. New insights into M1/M2 macrophages: Key modulators in cancer progression. Cancer Cell Int. 2021;21(1):389. 34289846 10.1186/s12935-021-02089-2 PMC8296555

[103] Wang Y, Li C, Wan Y, Qi M, Chen Q, Sun Y, Sun X, Fang J, Fu L, Xu L, et al. Quercetin-loaded ceria nanocomposite potentiate dual-directional Immunoregulation via macrophage polarization against periodontal inflammation. Small. 2021;17(41):2101505. 10.1002/smll.202101505 34499411

[104] Liu G, Xue J, Zhou X, Gui M, Xia R, Zhang Y, Cai Y, Li S, Shi S, Mao X, et al. The paradigm shifts of periodontal regeneration strategy: From reparative manipulation to developmental engineering. Bioact Mater. 2025;49:418–436. 40165829 10.1016/j.bioactmat.2025.03.009 PMC11957753

[105] Zhu X, Xiang D, Huo Y, He X, Chen F, Tian B, Li X. Progress in basic research and clinical strategies for cementum regeneration. Int Dent J. 2025;75(3):1566–1584. 40132248 10.1016/j.identj.2025.02.017 PMC11985013

[106] Zhao Z, Qiao Q, Sun Y, Wang J, Li X, Zhang L, Yang H, Zhang K, Zhang N, Bai Y. Human periodontal ligament stem cells and metformin to enhance periodontal regeneration in rats. J Dent. 2025;156: Article 105700. 40122368 10.1016/j.jdent.2025.105700

[107] Villagómez-Olea G, Uribe-Querol E, Marichi-Rodríguez FJ, Meléndez-Zajgla J, Alvaréz-Pérez MA, Rosales C. Periodontal ligament tissues support neutrophil differentiation and maturation processes. Front Immunol. 2024;15:1446541. 39588378 10.3389/fimmu.2024.1446541 PMC11586715

[108] Shakya A, Li Y, Chang N, Liu X. Supra-alveolar bone regeneration: Progress, challenges, and future perspectives. Compos Part B. 2024;283: Article 111673. 10.1016/j.compositesb.2024.111673 PMC11270636 39071449

[109] Nagata M, English JD, Ono N, Ono W. Diverse stem cells for periodontal tissue formation and regeneration. Genesis. 2022;60(8-9): Article e23495. 35916433 10.1002/dvg.23495 PMC9492631

[110] Zhu B, Wu J, Li T, Liu S, Guo J, Yu Y, Qiu X, Zhao Y, Peng H, Zhang J, et al. A glutathione peroxidase-mimicking nanozyme precisely alleviates reactive oxygen species and promotes periodontal bone regeneration. Adv Healthc Mater. 2024;13(4):2302485. 10.1002/adhm.202302485 37902093

[111] Sanz AR, Carrión FS, Chaparro AP. Mesenchymal stem cells from the oral cavity and their potential value in tissue engineering. Periodontology 2000. 2015;67(1):251–267. 25494604 10.1111/prd.12070

[112] Xie Y, Xiao S, Huang L, Guo J, Bai M, Gao Y, Zhou H, Qiu L, Cheng C, Han X. Cascade and ultrafast artificial antioxidases alleviate inflammation and bone resorption in periodontitis. ACS Nano. 2023;17(15):15097–15112. 37378617 10.1021/acsnano.3c04328

[113] Dong F, Liu Y, Dong H, Sun X, Xu B, Wu Q, Xu X, Jiang Y, Liu H, Wang Y. A nano-oxygen generator for enhanced light-controlled elimination of anaerobic bacteria in periodontal infections. Nano Today. 2024;56: Article 102280.

[114] Li X, Qi M, Li C, Dong B, Wang J, Weir MD, Imazato S, du L, Lynch CD, Xu L, et al. Novel nanoparticles of cerium-doped zeolitic imidazolate frameworks with dual benefits of antibacterial and anti-inflammatory functions against periodontitis. J Mater Chem B. 2019;7(44):6955–6971. 31617555 10.1039/c9tb01743g

[115] Wang Y, Chu T, Jin T, Xu S, Zheng C, Huang J, Li S, Wu L, Shen J, Cai X, et al. Cascade reactions catalyzed by gold hybrid nanoparticles generate CO gas against periodontitis in diabetes. Adv Sci. 2024;11(24):2308587. 10.1002/advs.202308587 PMC11199988 38647388

[116] Cai X, Jiao L, Yan H, Wu Y, Gu W, Du D, Lin Y, Zhu C. Nanozyme-involved biomimetic cascade catalysis for biomedical applications. Mater Today. 2021;44:211–228.

[117] Li L, Wu R-X, Wang J, Jin R, Zhang X-Y, Xu M, Li SY, Gao R, Tian BM, He XT, et al. A multirisk-rescued biomimetic nanozyme against periodontitis via inflammation targeting and microenvironment reprogramming. Chem Eng J. 2025;506: Article 160119.

[118] Huang X, Zhang S, Tang Y, Zhang X, Bai Y, Pang H. Advances in metal–organic framework-based nanozymes and their applications. Coord Chem Rev. 2021;449: Article 214216.

[119] Zhu C, Huang K, Li T, Li Y, Jin Y, Li R, Zhu Z, Yang S, Xia L, Fang B. Manganese dioxide coupled metal-organic framework as mitophagy regulator alleviates periodontitis through SIRT1-FOXO3-BNIP3 signaling axis. Biomaterials. 2025;319: Article 123179. 39983516 10.1016/j.biomaterials.2025.123179

[120] Li Y, Muhammad F, Chen X, Gu D, Li W, Tang J, Cheng M, du J, Qiao S, Deng Y, et al. Smart multifunctional Cu_2_O@RuO_2_ nanozyme for angiogenesis and osteogenesis in periodontitis. Nano Today. 2025;61: Article 102624.

[121] Li T, Shu M, Zhu C, Liu Q, Li Y, Wang R, Chen L, Shi W, Sun Z, Hou Z, et al. Triple-combination therapy with a multifunctional yolk–shell nanozyme Au@CeO_2_ loaded with dimethyl fumarate for periodontitis. Adv Sci. 2024;12(7):2413891. 10.1002/advs.202413891 PMC11831482 39716921

[122] Han Y, Yang L, Zhang J, Qu S, Wang Z, Gu B, Zhao Y, Zhang Y, Zhao Q, Ji Y. Circular RNA-chimeric CeO_2_ nanoplatform maintains protective autophagy in macrophages for periodontitis. Chem Eng J. 2025;503: Article 158338.

[123] Li S, Ding Q, Zhang L, Shi F, Liu C, Li T, Shi Y, Qi M, Wang L, Dong B, et al. Gold core@CeO_2_ halfshell Janus nanocomposites catalyze targeted sulfate radical for periodontitis therapy. J Control Release. 2024;370:600–613. 38735394 10.1016/j.jconrel.2024.05.016

[124] Fang J, Wang H, Bao X, Ni Y, Teng Y, Liu J, Sun X, Sun Y, Li H, Zhou Y. Nanodiamond as efficient peroxidase mimic against periodontal bacterial infection. Carbon. 2020;169:370–381.

