# Supplementary material for: Nanozymes Empower Periodontitis Treatment: New Strategies and Clinical Application Prospects
Source: Biomater Res. 2025 May 20;29:0210. doi: 10.34133/bmr.0210 (PMC12089970; doi:10.34133/bmr.0210)
Supplement: Supplementary 1 — Fig. S1 [file bmr.0210.f1.docx]

**
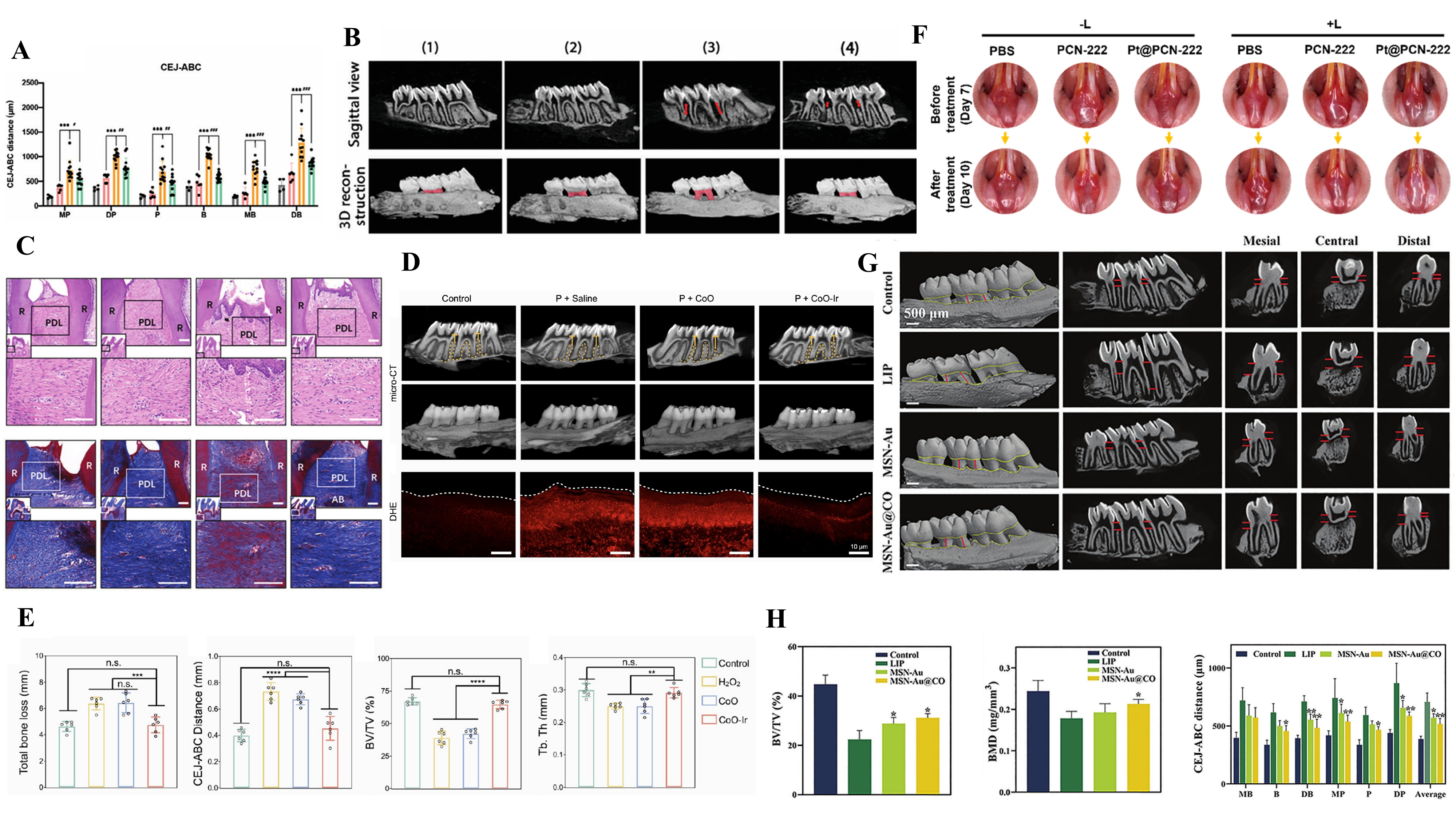
**

**Fig. S1.** (A): Statistical analysis of the Cemento-Enamel Junction to Alveolar Bone Crest (CEJ-ABC) distance. (B): Micro-CT analysis of periodontitis model after MVF treatment. (C): H&E staining of the periodontal tissues surrounding the maxillary second molar 30 days after MVF treatment. R: root. PDL: periodontal ligament; Masson staining of the periodontal tissues surrounding the maxillary second molar. (A, B, C) Reproduced with permission [118]. Copyright 2024, John Wiley and Sons. (D): Micro-CT analysis of periodontitis models after different treatments. (E): Statistical analysis of alveolar bone loss. Statistical analysis of the CEJ-ABC distance; Analysis of trabecular bone volume in the region surrounding the second molar. (D, E) Reproduced with permission [119]. Copyright 2023, ACS Publications. (F): Intraoral images of rats before and after different treatments. Reproduced with permission [120]. Copyright 2024, Elsevier. (G): Three-dimensional (3D) reconstructed digital images, mesial-distal cross-sectional images, and buccal-palatal cross-sectional images of M2 analyzed by Micro-CT. (H): Measure the bone volume (BV/TV), bone mineral density (BMD), and CEJ-ABC distance of M2. (G, H) Reproduced with permission [121]. Copyright 2024, John Wiley and Sons.
